# RNA-Seq Profiling Reveals Novel Hepatic Gene Expression Pattern in Aflatoxin B1 Treated Rats

**DOI:** 10.1371/journal.pone.0061768

**Published:** 2013-04-22

**Authors:** B. Alex Merrick, Dhiral P. Phadke, Scott S. Auerbach, Deepak Mav, Suzy M. Stiegelmeyer, Ruchir R. Shah, Raymond R. Tice

**Affiliations:** 1 Division of the National Toxicology Program, National Institute of Environmental Health Sciences, Research Triangle Park, North Carolina, United States of America; 2 Systems Research and Applications International, Durham, North Carolina, United States of America; University of Nevada School of Medicine, United States of America

## Abstract

Deep sequencing was used to investigate the subchronic effects of 1 ppm aflatoxin B1 (AFB1), a potent hepatocarcinogen, on the male rat liver transcriptome prior to onset of histopathological lesions or tumors. We hypothesized RNA-Seq would reveal more differentially expressed genes (DEG) than microarray analysis, including low copy and novel transcripts related to AFB1’s carcinogenic activity compared to feed controls (CTRL). Paired-end reads were mapped to the rat genome (Rn4) with TopHat and further analyzed by DESeq and Cufflinks-Cuffdiff pipelines to identify differentially expressed transcripts, new exons and unannotated transcripts. PCA and cluster analysis of DEGs showed clear separation between AFB1 and CTRL treatments and concordance among group replicates. qPCR of eight high and medium DEGs and three low DEGs showed good comparability among RNA-Seq and microarray transcripts. DESeq analysis identified 1,026 differentially expressed transcripts at greater than two-fold change (p<0.005) compared to 626 transcripts by microarray due to base pair resolution of transcripts by RNA-Seq, probe placement within transcripts or an absence of probes to detect novel transcripts, splice variants and exons. Pathway analysis among DEGs revealed signaling of Ahr, Nrf2, GSH, xenobiotic, cell cycle, extracellular matrix, and cell differentiation networks consistent with pathways leading to AFB1 carcinogenesis, including almost 200 upregulated transcripts controlled by E2f1-related pathways related to kinetochore structure, mitotic spindle assembly and tissue remodeling. We report 49 novel, differentially-expressed transcripts including confirmation by PCR-cloning of two unique, unannotated, hepatic AFB1-responsive transcripts (HAfT’s) on chromosomes 1.q55 and 15.q11, overexpressed by 10 to 25-fold. Several potentially novel exons were found and exon refinements were made including AFB1 exon-specific induction of homologous family members, Ugt1a6 and Ugt1a7c. We find the rat transcriptome contains many previously unidentified, AFB1-responsive exons and transcripts supporting RNA-Seq’s capabilities to provide new insights into AFB1-mediated gene expression leading to hepatocellular carcinoma.

## Introduction

Deep sequencing technologies provide unprecedented coverage of the transcriptome at nucleotide resolution and a wide dynamic range compared to hybridization microarrays based upon predefined probes [1], [2]. RNA-Seq offers the potential for *de novo* definition of intron-exon boundaries, 5′- and 3′-untranslated regions, splice variants, single nucleotide polymorphisms (SNPs), and potentially new transcripts at a highly accurate level of quantitation, all of which are crucial for the analysis of differential gene expression [3], [4], [5]. The laboratory rat is an important experimental animal model for the study of chemically-induced diseases but RNA-Seq studies of rat tissues [6], [7], [8], [9], [10], [11] are still rather limited in part because its complete genomic sequence and annotation are still being refined [12], [13]. Published rat transcript profiling studies have focused on effects in the ageing cerebral cortex [10], neurons in the nucleus accumbens [6], the hippocampus of alcohol-addicted rats [7], functional compartments in the rat placentation site [9], the ventricular myocardium from SHR rats, [8] and kidneys from aristolochic acid exposed animals [14]. Recent studies suggest that RNA-Seq is comparable to and provides a greater level of transcriptional detail than genome-wide microarrays, particularly for detecting low copy transcripts and that it provides for an overall higher dynamic range of signal intensity at 2 to 3 orders of magnitude greater than microarrays [14], [15].

Global gene expression studies using RNA-Seq can provide insights into regulatory genes and critical pathways that might lead to hepatocellular carcinoma [16], [17], [18]. For example, RNA-Seq of ten matched pairs of hepatocellular carcinoma and adjacent, non-cancerous tissues showed more than 1,000 differentially expressed genes and about 25,000 differentially expressed exons, including novel splice variants and a highly up-regulated exon-exon junction in the ATAD2 gene in HCC tissues [17]. In another study, CD90^+^ stem cells from human HCC and parallel non-tumorous liver tissue were cell sorted for deep sequencing of the transcriptome, revealing elevated glypican-3 among the 500 gene changes specific to liver stem cells [16]. Another genome-wide transcriptome survey in HCC patients identified recurrent hepatitis B virus (HBV) integration into sequences of induced, cancer-related TERT, MLL4 and CCNE1 genes [18]. Animal models can be valuable for studying underlying processes leading to HCC such as the aflatoxin B1 (AFB1) rodent model [19], [20] at 1 ppm in feed [21], [22] that involve metabolic activation to an 8,9-epoxide metabolite and which lead to DNA adducts, genetic damage, cellular transformation and HCC [23], [24]. Computational models have also been used to derive gene signatures from microarray data to distinguish genotoxic and non-genotoxic chemical agents prior to the onset of hepatic tumors, including HCC [25] and we recently validated one such signature for AFB1 [26].

The prospect of RNA-Seq’s increased resolution and sensitivity compared to microarray profiling suggests that the subchronic genotoxicity from AFB1 exposure might reveal new properties of the liver transcriptome not observable by conventional hybridization-based analysis. The goals of the current study were to more precisely define gene expression changes that might relate to carcinogenesis produced by AFB1 exposure prior to onset of malignancy and to begin a high resolution map of the F344/N rat liver transcriptome.

## Results

### Alignment of Sequencing Reads

All sequence data were at 2×75 bp length (Figure S1) with high quality metrics (>20 Phred score) and nucleotide distributions (Figure S2). The total number of sequenced reads ranged from 58–74 million pairs of which nearly 65% of the reads were uniquely aligned to the Rn4 genome assembly using the TopHat aligner (Table 1). The percentage of genomic alignment was similar between the two groups (CTRL, 64.3% ±0.4; AFB1 group, 65.0% ±0.6; mean±S.E.M) suggesting there were no obvious detectable biases in the sequence data. Alignment statistics indicated data were of high quality and were uniform (i.e., no outliers with reference to alignment proficiency) and provided sufficient sequencing depth to pursue differential expression testing between two groups.

**Table 1:** Alignment of RNA-Seq Reads to the Rat Genome^a^.

Sample	Total reads	Total (uniquely) aligned reads	% Aligned	Both Ends mapped	Singletons	Spliced reads
CTRL_0	67,395,930	43,936,057	65.19	37,125,804	6,810,253	9,294,996
CTRL_1	64,538,856	41,448,207	64.22	35,167,586	6,280,621	10,012,853
CTRL_2	74,002,372	46,839,123	63.29	39,886,516	6,952,607	10,501,161
CTRL _3	71,263,544	46,051,262	64.62	39,785,288	6,265,974	9,963,622
AFB1_0	59,987,334	37,890,030	63.16	31,823,402	6,066,628	7,649,739
AFB1_1	58,988,872	38,868,457	65.89	33,733,256	5,135,201	9,106,175
AFB1_2	60,843,500	40,205,023	66.08	35,058,722	5,146,301	8,876,374
AFB1_3	66,458,154	43,107,025	64.86	37,343,234	5,763,791	9,919,540

a Paired-end reads were aligned to Rn4 using TopHat v1.3.2 and unique, non-gapped alignments were obtained. TopHat parameters were: -g 1–r 200–best –strata, where ‘-best’ and ‘–strata’ are bowtie parameters that were included in the TopHat source code. Each sample represents an individual RNA-Seq lane of liver mRNA from one animal. The last three columns indicate the number of reads in which both ends were mapped; only one end was mapped but not both (singletons); and reads that were spliced to allow for alignment, respectively.

### Transcript Assembly and Differential Expression Analysis

The Cufflinks pipeline was used to assemble transcripts and to estimate their abundance. After assembling transcripts individually for each sample, we employed Cuffcompare to produce a union of transcripts from all eight samples which yielded 57,076 transcripts (Table 2). Of all the transcripts assembled, 14,257 completely matched RefSeq transcript annotations and over twice that number (30,877) partially matched RefSeq annotation. Over eleven thousand transcripts were obtained which likely included novel transcripts. The number of RNASeq transcripts assembled by Cufflinks that matched (overlapped) microarray probe sets were 44,469 for which 85.9% (38,186 transcripts) had RefSeq annotation, while the remaining 14.1% (6,283 transcripts) were classed as potentially novel (Table 3). Interestingly, 12,607 more total transcripts were found by RNA-Seq (18.2% more than microarray) and an additional 5,649 transcripts were classified as novel since they did not overlap a microarray probe or RefSeq annotation. Our findings suggest that RNA-Seq identified almost double the number of unannotated transcripts compared to microarray analysis.

**Table 2:** Reference-Guided Assembly of RNA-Seq Reads into Transcripts by Cufflinks^a^.

Total Transcripts Assembled	57,076
Complete Match, RefSeq	14,257
Partial Match, RefSeq	30,887
Novel Candidates	11,932

a Cufflinks was employed to assemble reads into transcripts from each animal sample and Cuffcompare was used to obtain a union of transcripts from all 8 animals as summarized in this panel. ‘Total Transcripts Assembled’ was the sum of Complete, Partial and Novel transcripts. ‘Complete Match’ transcripts were identical to RefSeq transcripts (intron chain matches RefSeq gene); transcripts in the ‘Partially Match’ category include transcripts that overlap RefSeq transcripts but do not completely match (these could include potentially novel isoforms of an existing RefSeq transcript); and potentially ‘Novel’ transcripts are those that did not overlap with any RefSeq transcripts at all and may therefore involve intergenic transcripts.

**Table 3:** Analysis for Differentially Expressed Genes (DEGs)^a^.

Transcripts by Analysis	TotalAssembled	RefSeq	Novel
Total CuffCompare(− ‘Others’)	57,076	45,144	11,932
Cufflinks (MA probe+RNASeq)	44,469	38,186	6,283
DESeq DEG^(2X Fold, p<0.005)^	1,026	945	81
Microarray (MA) DEG^(2X Fold,^ ^p<0.005)^	626	558	68
Cuffdiff DEG^(2X Fold, p<0.005)^	119	108	11

a Transcripts were grouped by various combinations of analysis into three columns: Total Assembled transcripts; RefSeq - transcripts matching or partially matching RefSeq genes; and Novel transcripts. In the first row, Total CuffCompare transcripts included all transcripts (57,076); RefSeq transcripts (complete or partial match) were 45,144 (14,257+30,887 = 45,144); and 11,932 potentially novel transcripts. Note, that a set of 1,496 Cufflinks assembled transcripts referred to as, ‘Others’, contained significant repeat sequences and so this small set of transcripts was excluded (e.g. –‘Others’) from Total Assembled transcripts. In row 2, to enable comparison with available Microarray data, we identified Cufflinks assembled transcripts that overlapped MA probes (total 44,469 transcripts). From this group of transcripts, we determined differential expression (DEGs) using student’s t-test in the case of MA (microarray) data in row 4, or for RNA-Seq data we performed DESeq analysis in row 3 or Cuffdiff analysis in row 5 (see Materials and Methods for details).

Comparison of Cufflinks transcript level signals among the four samples within the CTRL or AFB1 treatment groups showed a high degree of correlation (Figure S3) with correlation coefficients (r^2^) uniformly about 0.98. Similarly, high r^2^ values (0.96–0.99) were observed for normalized probe intensities for microarray data between samples for each treatment group (Figure S4). The results of these analyses indicate that hepatic gene expression of rats that were measured by RNA-Seq and microarray platforms were highly stable and reproducible.

DESeq and Cuffdiff were used to test for differential expression on Cufflinks assembled transcripts. Among the large number of expressed liver transcripts, our initial differential expression analysis focused upon changes in well annotated genes from RefSeqGene as defined by RefSeq annotations. RefSeqGene is a subset of NCBI’s Reference Sequence (RefSeq) project and defines genomic sequences that have sufficient literature support as reference standards for well-characterized genes that generally represent a prevalent, ‘standard’ allele. For differential expression of RNA-Seq data, we used Cufflinks for transcript assembly followed by Cuffdiff or DESeq for their different capabilities in measuring and comparing transcript changes between groups. RNA-Seq and microarray data were generated from the same RNA sample for each animal to permit direct comparison of differential expression from two high throughput platforms. Correlation analysis of Cufflinks transcript expression (RNA-Seq) and normalized probe intensities (microarray) were performed by first dividing genes into quartiles, based on their expression levels in control conditions, and then correlating expression measurements originating from either RNA-Seq or microarray platforms. A correlation was performed for all animal samples from CTRL (Figure S5) and for AFB1 (Figure S6) treated animal samples. As expected, the highest correlations were found for highly expressed transcripts in quartile 4, with a comparatively weaker correlation among transcripts in the lower quartiles. These data indicate that a better correlation occurs between RNA-Seq and microarray platforms as signal level increases.

**Figure 1:**
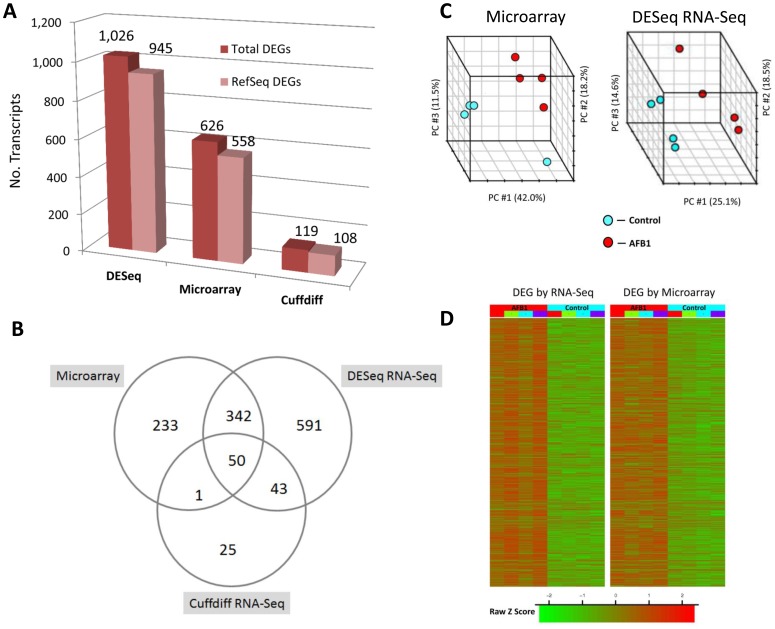
Differentially expressed genes (DEGs) identified from RNA-Seq by DESeq and Cuffdiff compared to microarray. Panel A. Number of DEGs observed with change ≥2-fold at p≤0.005 by DESeq, microarray and Cuffdiff analyses with Cufflinks assembled transcripts for all eight animals (4 Control, 4 AFB1) is shown. Total number of Cufflinks assembled transcripts and those that match an existing RefSeq annotation are displayed in the bar chart. Panel B. Venn diagram of DEGs from Panel A shows the number of common transcripts (overlapping circles) and unique transcripts (non-overlapping circles) for all three analyses. Panel C. Principal component (PC) analysis was performed for all samples using the gene expression values for DEGs found by microarray and DESeq (on Cufflinks assembled transcripts) analysis. The percentage variability captured by the first three principal components is displayed across PC#1, 2 and 3 represented on X, Y and Z axes. Panel D. Heatmap shows all DEGs at ≥2-fold change, p≤0.005 from microarray or DESeq analyses (on Cufflinks assembled transcripts). Gene expression data were log2 transformed and then quantile normalized prior to generating the Heatmap for direct comparison of data. DEGs (red or green are upregulated or downregulated, respectively) for each animal were mapped by lane for each of four animals in the CNTL or AFB1 treatment groups.

The number of DEGs obtained at a 2-fold cutoff and unadjusted p<0.005 from RNA-Seq data by Cuffdiff and DESeq were compared to microarray profiling (Figure 1A). DESeq identified 1,026 DEGs which were 8.6 times more than the 119 DEGs identified by Cuffdiff and almost twice the 626 DEGs detected by microarray analysis. Among the DEGs, about 90% of the transcripts were annotated by RefSeq for each of the DESeq, Cuffdiff and microarray analyses, leaving approximately 10% that were potentially novel, differentially expressed transcripts. Venn diagram analysis of DEGs (Figure 1B) shows the common and unique transcripts shared among the three analyses for DEGs from RNASeq (DESeq and Cuffdiff) and Microarray platforms. Unique and common transcripts from the Venn diagram are summarized in Table S1. The top 30 expressed transcripts among the three platforms are provided in Table S2. The proportion of DEGs that were shared between RNA-Seq and microarray varied by analysis; 62.6% (392/626 transcripts) for DESeq and 8.1% (51/626 transcripts) for Cuffdiff analysis. Reasons for varying differential expression by Cuffdiff compared to DESeq likely relate to the depth of sequencing, unique distribution of genes and their expression levels in liver tissue and fragment alignment cutoffs for statistical comparison of low expression genes [27]. As a result of greater DEGs, we focused on DESeq transcripts for further comparison to microarray data. The PCA plot showed clear separation between CTRL and AFB1 treated groups (Figure 1C). Cluster analysis of all samples using DEGs (Figure 1D) shows a similar increase and decrease in transcript expression produced by AFB1 exposure compared to CTRL as well as a high degree of intragroup homogeneity of expression for both DESeq and microarray analyses. We note that in comparing expression platforms, transcript normalization was uncorrected for length since we computed fold differences for each corresponding transcript in CTRL and AFB1 groups and not across different transcripts. When RPKM normalization for RNA-Seq data was computed and compared to microarray data (Table S3) we found results were similar to those presented in Figure 1.

### Validation of DEGs by qPCR

**Figure 2:**
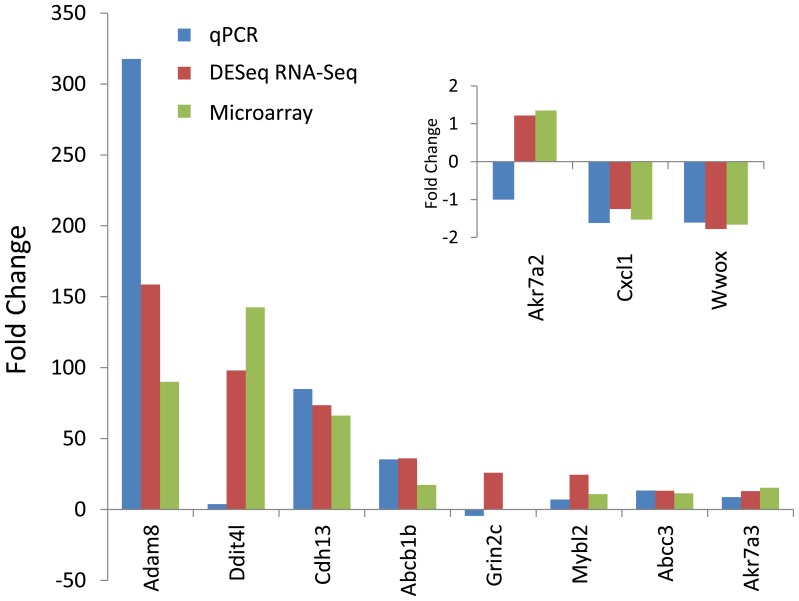
Test and validation of differential expression between qPCR, RNA-Seq and microarray by select transcripts. The mean fold changes for each group were compared in the bar chart for eight high to medium fold change transcripts and three low expression change transcripts (inset). qPCR data were normalized to β-actin expression for each sample. Means of significant (p≤0.05) fold changes from control were computed for qPCR, DESeq and microarray using RNA from the same 4 animals in each analysis.

Expression changes of selected high, intermediate or low expressing transcripts were analyzed by qPCR as described previously [26] and compared to the same samples analyzed by microarray and DESeq (Figure 2). Among genes displaying large differential expression changes, Adam8 and Cdh13 were in good agreement for consistency of response. Ddit4l increased from 98-fold to 142-fold fold by DESeq and microarray, respectively, but did not amplify well by qPCR (3.8-fold) which may be related to sequence specific issues and primer selection. Genes with intermediate expression agreed well among four of five transcripts (Abcb1b, Mybl2, Abcc3, Akr7a3) while Grin2c was slightly reduced at −4.5-fold by qPCR compared to no change by microarray and a 25-fold increase observed with DESeq. For low expression change transcripts, two (Cxcl1 and Wwox) of three transcripts agreed in direction and relative magnitude for these three platforms with the only exception being Akr7a2 at (−1)-fold by qPCR compared to slight increases at 1.2-fold and 1.3-fold in DESeq and microarray analyses, respectively. Primer design and other experimental conditions are probably responsible for some varying expression results by qPCR with the other platforms for these few transcripts as previously described [26]. Overall, a comparison of a wide range of DEGs shows validation of the direction of change for 9 of 11 transcripts analyzed by RNA-Seq and microarray.

### Greater Number of DEGs by RNA-Seq

**Figure 3:**
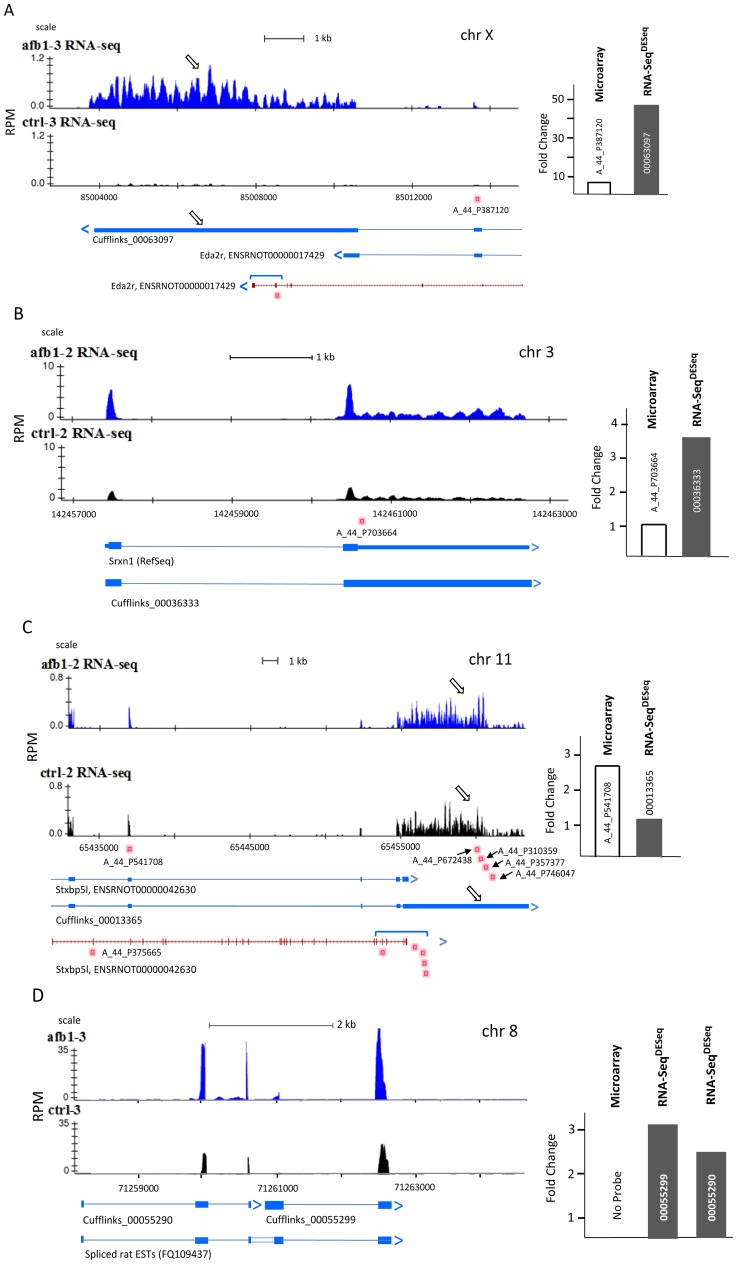
Differing types of DEGs found by RNA-Seq and microarray data (Panels A–D). Numbers of reads in RPM (reads per million mapped reads) are shown on the Y-axis and the genomic region is displayed on the X-axis using a common NCBI scale for representative AFB1 (blue reads) and CTRL (black reads) sample tracks in UCSC browser format. Placements of microarray probes for specific transcripts are indicated by small rose-colored squares with probe names below. Numbered Cufflinks assembled transcripts and the corresponding RefSeq (gene abbreviation) or Ensembl annotated transcripts (ENSRNOT) identifiers are displayed under the RNA-Seq tracks. Exons are represented as blocks or bands; introns are lines between exons. Arrows at the end of assembled or annotated transcripts show the direction of transcription. Open arrows in specific panels point out a new Cufflinks identified exon. Bar graphs to the right show mean fold changes (AFB1/CTRL) for specific microarray probes and the corresponding RNA-Seq transcripts (DESeq). At the bottom of Panels A and C, the entire transcript (red) is presented for which a portion of the transcript which has been enlarged (blue bracket) for the RNASeq tracks shown above. In Panel D, only spliced EST’s (parallel lines in the center of spliced EST represent gaps in the alignment) were available which were without RefSeq or Ensembl transcript annotation and for which no microarray probe had been assigned. In Panel D bar graph, fold changes for two separate Cufflinks transcripts are indicated while no microarray data were available (no probe).

We were interested in possible explanations of why RNA-Seq data showed an increased number of DEGs compared to microarray (Figure 1A). Investigation into several example cases, as described in the gene tracks in Figure 3, revealed several possible explanations that generally involved greater nucleotide level resolution data provided by RNA-Seq analysis which allows for both accurate quantification of expression levels and precise demarcation of exon boundaries compared to probe hybridization. For example, Eda2r (ectodysplasin A2 receptor) is a plasma membrane bound receptor regulating initiation, morphogenesis and differentiation of ectodermally-derived organs [28]. Figure 3A shows a novel extension at the 3′-end of the Eda2r transcript (open arrows) as assembled by Cufflinks. These additional reads observed in an assembled transcript, Cufflinks_00063097, are responsible for the larger fold increase at 45.5-fold by DESeq and are not reflected by the smaller increase of 6.3-fold of single probe (A_44_P387120) data at the 6^th^ exon. It appears likely that these additional reads in RNA-Seq comprise an undescribed UTR (observed continuity of reads, no exon breaks) portion of the Eda2r transcript but additional work will be needed to support this hypothesis. Figure 3B shows how probe placement can affect which genes are identified as differentially expressed between platforms. The probe, A_44_P703664, for the RefSeq transcript of Srxn1 (sulfiredoxin 1; GSH-depletion related oxidative stress [29]) which was placed in the 3′-region (3′-end microarray probe design [30]) detects no significant change at 1.4-fold from AFB1 treatment. However, DESeq measured a 3.5-fold increase for a transcript (Cufflinks_00036333) which represents expression of all exons and UTRs that are identical to the RefSeq transcript (NM_001047858) for Srxn1. Similarly, different results are indicated by microarray probe and DESeq fold changes in Figure 3C. The bar graph (Figure 3C) shows a 2.7-fold increased hybridization to probe A_44_P541708 for Stxbp5L (Syntaxin-binding protein 5-like; vesicle trafficking, exocytosis, negative regulator of insulin secretion [31]) but DESeq for the Stxbp5L transcript shows no significant difference at 1.2-fold change (bar graph) when all exons, including a novel 3′- region (open arrows), are counted. Interestingly, no fold change was found for the 5′-end probe A_44_P375665 (see whole transcript at bottom of figure) nor were any significant fold changes observed for four additional microarray probes located within the 3′-terminal region (not yet annotated to RefSeq Stxbp5L or Ensembl Stxbp5L transcript). We further note that no Cufflinks transcripts included the 1^st^ exon of Stxbp5L (consistent with no observed reads in exon 1; data not shown) suggesting transcription began at the 2^nd^ exon in liver tissue. In Figure 3D, novel Cufflinks transcripts which were significantly upregulated (3.2-fold increase for Cufflinks_00055299; and 2.5-fold increase for Cufflinks_00055290) did not have any probes assigned to these transcribed locus regions (spliced ESTs) in chromosome 8. Potentially, these two adjacent Cufflinks transcripts (Cufflinks_00055299 and Cufflinks_00055290) represent different portions of one transcript but this awaits experimental confirmation.

### Novel Transcripts Found by RNA-Seq

**Figure 4:**
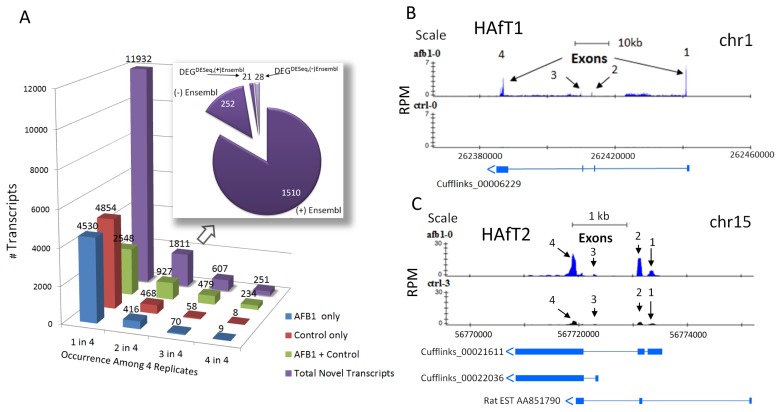
Novel transcripts found by RNA-Seq. Panel A. Bar graph shows the number of total and potentially novel transcripts on the Y-axis. Transcripts are shown for Total (purple), unique to either AFB1 (blue) or Control (red), and common (AFB1+ Control, green), according to the occurrence among replicates on the X-axis. A further breakdown of the 1811 total transcripts observed in two or more replicates is shown in the pie chart. Here, the majority of 1811 transcripts had some Ensembl annotation (1531) while the rest did not (280). Of those that were differentially expressed by AFB1, 21 had Ensembl annotation while 28 were unannotated and therefore, novel DEG transcripts. Panels B and C show two such novel transcripts, described here as ‘HAfT-1’ and ‘HAfT-2’ (Hepatic Aflatoxin-responsive Transcripts 1 and 2). Numbers of reads as RPM are shown on the Y-axis and the genomic region is displayed on the X-axis for representative AFB1 (blue reads) and CTRL (black reads) sample tracks in UCSC browser format. See text for more details.

Of the 57,076 total transcripts in Table 2 that were assembled from all eight animals, 11,932 (or 20.9%) were considered novel if they were outside of RefSeq gene annotations; novel transcripts could include Ensembl transcripts (ESTs, transcriptionally active loci, hypothetical transcripts) or transcripts without prior annotation. To ensure reproducibility, we counted how many animal replicates contained each of the novel transcripts and selected those transcripts that appeared in more than one replicate (Figure 4A). Additionally, we also determined if the novel transcripts appeared in either CTRL or AFB1 treatment conditions or both. As expected, the number of total novel transcripts diminished as the criteria for reproducibility increased. A further breakdown of the 1,811 potentially novel transcripts observed in more than 50% of the animals (two or more rats) is shown in the inset of Figure 4A. In the group of 1811 Cufflinks transcripts, there were 1532 (+) that were also annotated in Ensembl (1510 transcripts at no change and 21 transcripts with differential expression), and 280 transcripts for which there were no (−) Ensemble annotations (252 transcripts with no change and 28 transcripts with differential expression). Only 2.7% or 49 novel transcripts met criteria for differential expression by DESeq (≥2-fold, p<0.005) of which 21 transcripts had some (+) Ensembl annotation and 28 transcripts, shown as (−) Ensembl, which did not have an Ensembl annotation (Table S4).

A particularly interesting subset of these novel transcripts, DEG^DESeq, (−)Ensembl^, were those unique DEGs responding to AFB1 but without rat RefSeq/Ensembl annotation. We have termed two such Cufflinks assembled transcripts as ‘HAfT’ or ‘Hepatic Aflatoxin-responsive Transcripts’ (Figures 4B, 4C). The HAfT’s were further confirmed with independent validation by Sanger sequencing. The HAfT1 transcript was assembled as 4 exons (Cufflinks_00006229) in Chr1.q55 (Figure 4B). HAfT1 was found in all four animal samples with an average upregulation of 21.5-fold in AFB1 treated rats compared to CTRL and is located in an intergenic region between rat Tcf7l2 (NM001191052) and Habp2 (RGD:1302979). Comparative genomics shows this intergenic region in the rat corresponds to the first intron of the homologous mouse Tcf7l1 gene (NM009332) in which HAfT1 is in an antisense orientation. No prior ESTs correspond to HAfT1. Primers were designed from the sequence of the Cufflinks transcript to clarify the exons of HAfT1. Sequencing of cloned PCR products from two AFB1 rats (AFB1-0 and AFB1-1) showed an 809 bp cDNA that aligned with predicted exons (Figure S7A). A hypothetical peptide sequence of 115 amino acids could be translated from a putative ATG start site at position 31 before encountering a stop codon. Preliminary analysis suggests this novel AFB1-responsive gene may be protein coding although further experiments will be needed to demonstrate a translation product. A second transcript, HAfT2, is also reported in Figure 4C as a proposed 4 exon transcript (a combined product of Cufflinks_00021611 and Cufflinks_00022036) on Chr15.q11. The Cufflinks_00021611 transcript was upregulated an average of 8.5-fold by AFB1 treatment compared to the CTRL group. In this case, two exons of rat EST AA851790 partially overlapped with the Cufflinks transcripts. We note that there was no evidence of any reads from our RNA-Seq data (Figure 4C) to support the presence of the first designated exon in the EST AA851790 (in liver tissue). We cloned PRC products from the same AFB1 treated rats as above after designing primers for the 4^th^ exon. The 209 bp cDNA aligned well with the last exon of this new AFB1 inducible transcript (Figure S7B) that was 8 kD downstream of the 5′-end of the rat Tpt1 gene (RGD:621623). Additional work must be performed to confirm the sequence and expression of the entire transcript proposed for HAfT2; however, our computational predictions followed by PCR-sequencing validations strongly support the presence of these novel HAfT1 and HAfT2 transcripts in liver and for their upregulation by AFB1 exposure.

### RNA-Seq Reveals Novel Exons

**Figure 5:**
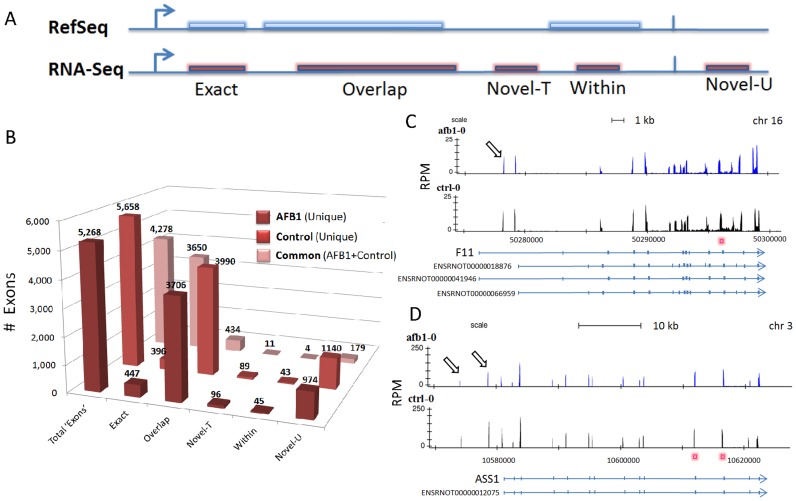
Novel exons found by RNA-Seq analysis. Panel A shows classification of different types of exons encountered during analysis of a Cufflinks assembled transcript in a model gene. Exons include ‘Exact’ matches to known exons, ‘Overlapping’ exons corresponding to partial matches, ‘Novel-T’ exons that occur with known transcripts, ‘Within’ exons occurring within the sequence of known exons and Novel-U exons that were unknown and occur outside known transcripts. Panel B is a bar chart of such exon types that were unique to AFB1 or Control treatments and those exons that were shared between treatments. Examples are shown for a Novel-T exon (Panel C) within the F11 transcript and two Novel-U exons (Panel D) outside the ASS1 gene. Numbers of reads, as RPM, are shown on the Y-axis and the genomic region is displayed on the X-axis for representative AFB1 (blue reads) and CTRL (black reads) sample tracks in UCSC browser format.

Another goal of this study was to compare all exons generated during the transcript assembly process and classify them into exon categories in order to identify novel or newly annotated exons (Figure S8). Our approach was to compare Cuffcompare transcript fragments for each treatment with the RefSeq annotation and place transfragment exons into various categories according to the schema in Figure 5A. Only those exons were considered that passed selection criteria for statistical significance of expression were used in this analysis as described in Materials and Methods. A total of 15,204 total exons met stringent criteria for consideration and about one-half of the total exons were common (4,278 exons) to either control (9,936 Total CTRL exons) or AFB1 (9,546 Total AFB1 exons) treatment groups in (Figure 5B). Most of the exons common to both groups were Exact matches to RefSeq exons (85% of Common, Total ‘Exons’) while the other groups comprised smaller proportions (Common Novel T, Common Novel-U locations shown in Table S5). Interestingly, ‘Overlap’ exons made up the largest share of exons unique to CTRL (71%) and unique to AFB1 (70%), followed by about 20% of Novel-U exons which were found outside known transcripts. ‘Exact’ matching exons, Novel-T exons, and ‘Within’ exons made up about 10% of the total exons unique to either CTLR or AFB1 groups. Overall, RNA-Seq analysis found almost two hundred Common novel exons either within the positional boundaries of annotated transcripts or outside RefSeq annotation (genomic locations of novel exons are described in Table S5). These novel exons from RNASeq data await further experimental validation.

A few examples of Novel-T, Novel-U and Overlap exons are presented in Figures 5C, 5D and 3A and 3C. RefSeq and Ensembl annotations are shown in Figure 5C for the F11 gene (coagulation factor XI) for which a Novel-T exon was found by Cufflinks in both treatment groups. It seems reasonable that this transcript could include an alternative start site for F11 since this Novel-T exon appears downstream of the predicted first exon (no reads visible for predicted exon 1). Human F11 is recognized for alternative splicing events in liver and platelets [32]. Extensions of existing annotation at 3′-exons were described for Eda2r in Figure 3A that represent an Overlap exon and also for Stxbp5l (Figure 3C) that represent a Novel-U exon as one outside the RefSeq and Ensembl annotated boundaries. A more extensive example of multiple Novel-U exons is shown in Figure 5D for the two exons (open arrows) toward the 5′-end of the Ass1 (argininosuccinate synthase 1) gene, a well-expressed intermediary metabolism enzyme (Figure 5D). In addition, we also provide eight additional examples in Figure S9A to S9H of novel exons discovered within known RefSeq or Ensembl gene boundaries that are conserved in other species (e.g. human, mouse) but are not yet annotated in rat.

**Figure 6:**
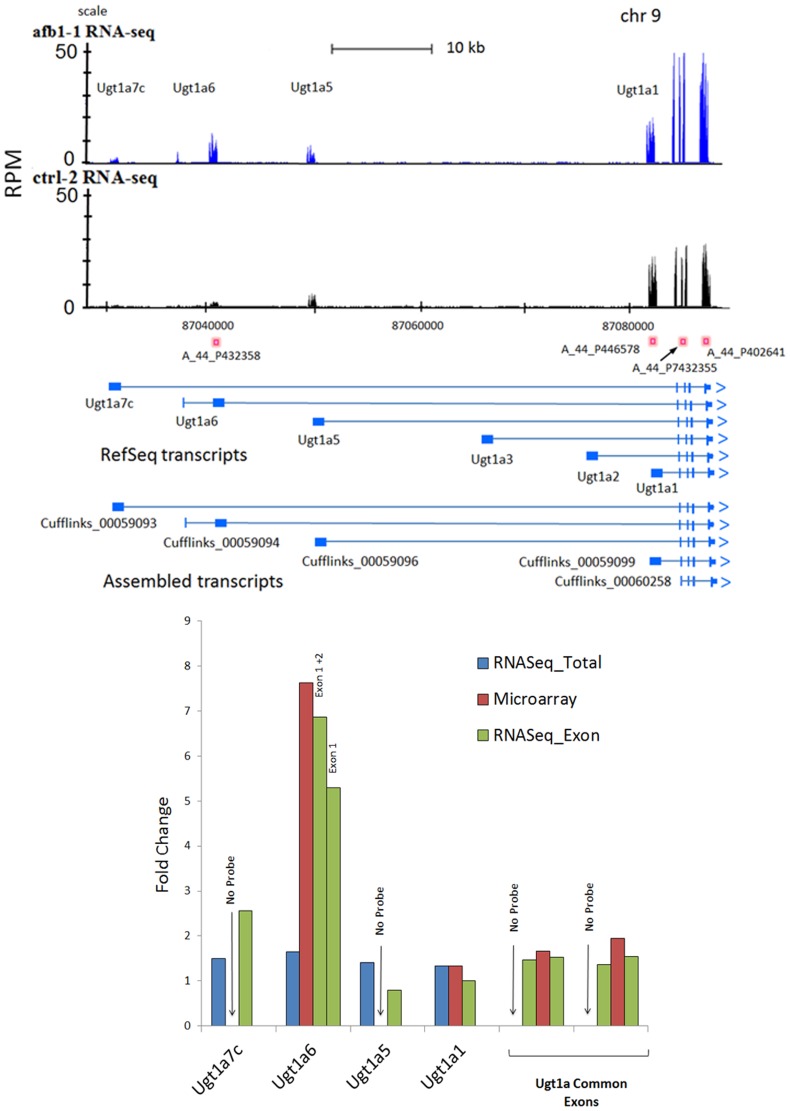
Exon specific expression among homologous transcripts in the Ugt1a gene family. Panel A. The genomic region for Ugt1a transcripts is displayed on the X-axis in UCSC browser format where the Y-axis represents mapped reads in RPM units. Placements of microarray probes for specific transcripts are indicated by rose-colored boxes with probe names below. There are a total of four microarray probes, some of which correspond to shared exons of the Ugta1 gene family (A_44_P432355, A_44_P402641) or to specific exons defining Ugt1a1 (A_44_P446578) and Ugt1a6 (A_44_P432358) isoforms. RefSeq transcripts and Cufflinks assembled transcripts are displayed under the RNA-Seq tracks. Exons are shown as light blue blocks or bands; introns are lines between exons; arrows at the end of each transcript indicate direction of transcription. Panel B. Bar graph shows mean fold changes (AFB1/Control) on the Y axis for the entire RNA-Seq transcript (blue), the microarray probe (red) and the isoform-specific RNA-Seq_exon (green). For some exons, there was no corresponding microarray probe − ‘No Probe’ (e.g. Ugt1a5, Ugta1a7c). Exon-specific, RNA-Seq ratios were labeled by exon number. Ugt1a-Common consists of four exons (common to all Ugt1a isoforms) for which two microarray probes exist. Exon-specific ratios from RNA-Seq reads were calculated for Exons 1, 2, 3 and 4. RNA-Seq exon-specific reads were measured to calculate AFB1/Control ratios for Ugt1a1, Utgt1a5, both exons of Ugt1a6, and Ugt1a7c.

The preceding data demonstrate the ability of RNA-Seq in expanding the current annotation of the rat transcriptome at both the transcript and exon level. However, alternative gene isoforms present certain challenges to both RNA-Seq and microarray for the accurate quantitation of transcript expression levels and the ability to distinguish one isoform from another. We explored such a case for Ugt1a transcripts, belonging to the UDP glucuronosyltransferase 1 family, polypeptide A cluster, which are actively expressed phase II, conjugators of xenobiotics and relevant for AFB1 toxicity [33]. Ugt1a isoforms share the last four exons but generally contain one unique 5′-exon to define each isoform. RefSeq annotation currently contains eight isoforms for this gene family, namely, Ug1a1, Ugt1a2, Ugt1a3, Ugt1a5, Ugt1a6 and Ugt1a7c, Ugt1a8 and Ugt1a9. Figure 6A shows assembled transcripts for Ugt1a1 isoforms except for Ugt1a8 and Ugt1a9 which were not expressed. Expression of the four common exons at the 3′-end of Ugt1a gene family is similar in CTRL and AFB1 samples which is evident from the fold change observed for the two microarray ‘Ugta1a-Common’ probes as well as the fold change measured by RNA-Seq for each of the individual exons (Figure 6B). Expression changes of AFB1 vs. CTRL were about two-fold for all Ugt1a isoforms using RNA-Seq primarily because the four common exons contribute the majority of reads mapped to each isoform, potentially masking any fold change observed in isoform specific 5′-exon(s). For example, AFB1 specific induction of the two unique exons for Ugt1a6 isoform in AFB1 was not observed when ratios were calculated for the whole transcript. By contrast, the microarray probe annotated specifically for Ugt1a6 isoform was elevated by 7.3 fold. Only when exon specific reads from RNA-Seq data were counted for isoform specific exons of Ugt1a6 (exons 1 and 2) were the fold change values found to be comparable (induction of 6.7 and 5.4 fold, respectively). On the other hand, RNA-Seq analysis provided clear evidence for expression of both Ugt1a5 and Ugt1a7c, whereas no microarray data were available because microarray probes were absent for these genes. RNA-Seq exon specific reads showed a 2.7 fold increase for Ugt1a7, though this was not statistically significant. Thus, exon specific data from RNA-Seq reads or microarray probes placed at isoform defining exons appear best qualified to give credible isoform measurements among homologous family members.

### Activation of Biological Pathways by AFB1

**Figure 7:**
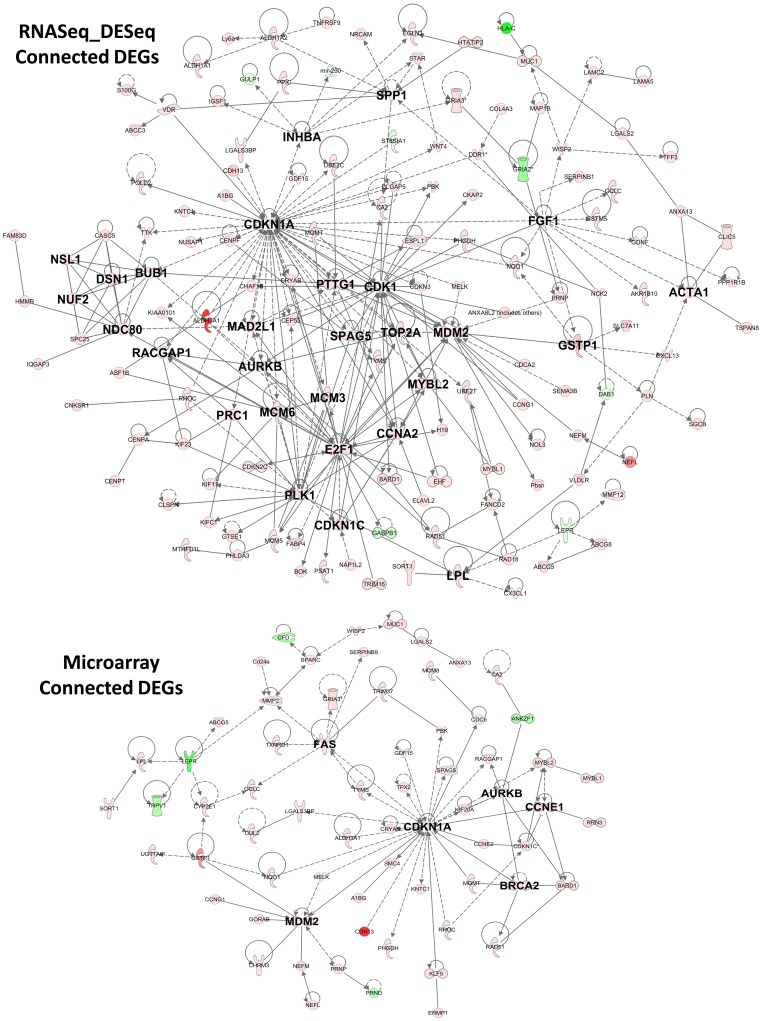
Connection pathway analysis of DEGs from subchronic AFB1 exposure. The top panel shows annotated interactions and regulatory relationships using IPA’s (Ingenuity Pathway Analysis) connectivity analysis. The connective pathway maps were generated using DEGs identified by DESeq_RNASeq (top panel) and for DEGs generated from microarray analysis (bottom panel) for only those transcripts with available RefSeq annotation. Hub genes (bolded, enlarged gene symbols) were defined as those transcripts regulating or interacting with ≥5 transcripts (red, upregulated; green, down-regulated).

The biological effects of AFB1 exposure prior to development of hepatic tumor formation are of interest to better understand malignant transformation by this model carcinogen. Pathway analysis assisted us in determining the biological effects of AFB1 exposure using genes with statistically significant differential expression. DEGs from AFB1 exposure were contained in various canonical pathways for which 26 were common (Table S6) to the three approaches used for differential expression. Representative pathways involved xenobiotic metabolism (AhR and xenobiotic metabolism signaling, glutathione metabolism), cell cycle dysregulation (G1/S checkpoint regulation, cyclins and cell cycle, ATM signaling, estrogen-mediated S-phase entry), malignancy pathways (glioma and hereditary breast cancer signaling), Nrf2-mediated oxidative stress, and ten various intermediary and amino acid metabolism pathways. Since the categories of canonical pathways found by RNA-Seq platforms and microarrays were similar, we considered the type and number of interrelationships among DEGs datasets as another means of identifying potential drivers of biological alterations that could set the stage for AFB1 carcinogenesis. Twenty-six transcripts showed substantial connections from DEGs by DESeq (Figure 7A). Here, Cdkn1a, E2f1, Cdk1, Mdm2, Fgf1, Ndc80, Bub1, Ccna2, Aurkb, Pttg1 and Spp1 appeared to be major interacting transcripts showing 46, 28, 27, 14, 12, 10, 9, 8, 8, 8 and 8 connections (Table S7), respectively. A number of elevated transcripts also formed mutual connections relating to kinetochore structure and supportive functions including Bub1, Ndc80, Mad2L1, Nsl1, Aurkb, Nuf2, Dsn1, Mcm3 and Mcm6 (Table S7). Microarray DEGs had far fewer connections at 6 transcripts; Cdkn1a, Mdm2 and Fas were primary contributors at 25, 8 and 8 connections, respectively (Figure 7B, Table S8). Only four substantial connected transcripts were observed for Cuffdiff (Cdk1, CCnd1, Cdk1 and Fas had 24, 21, 14 and 5 respective connections; Table S9). Among these pathway connection maps in Figure 7A and 7B, changes in transcripts responsive to DNA damage were insufficient to form a connection hub even though there were upregulated transcripts (2 to 4-fold increase) associated with DNA damage and repair processes including, Mgmt, Top2a, Rad51, Rad18, Xrcc6, Mnd1 and Tyms.

**Figure 8:**
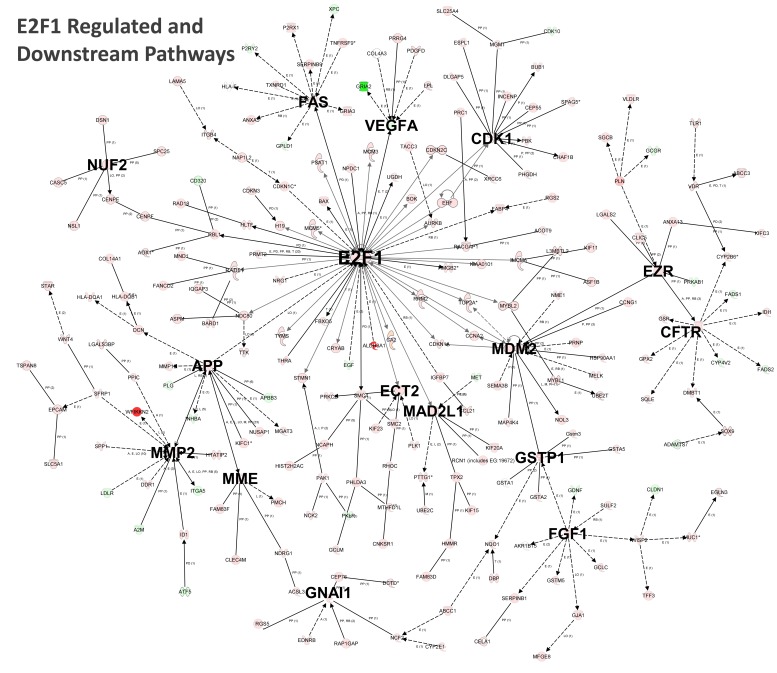
E2f1 regulated and downstream pathways altered by AFB1. AFB1 produced DEGs from DESeq analysis of RNA-Seq data which were analyzed by the IPA’s ‘grow pathway’ analysis (Ingenuity Pathway Analysis) which displays annotated regulatory relationships and interactions. Starting with induction of E2f1 in the center, DEGs from DESeq analysis were used to connect and grow downstream-dependent genes (red, upregulated; green, down-regulated). Hub genes (bolded, enlarged gene symbols) were defined as those transcripts regulating or interacting with ≥5 transcripts.

Several of the interacting transcripts in Figure 7 are involved in enhanced cell proliferation and turnover including E2f1, a member of the E2f family of transcription factors, which was upregulated by AFB1. Among many other cell cycle genes, E2f1 plays a critical role in controlling both cell cycle progression and apoptotic cell death in response to DNA damage (e.g. hepatic metabolites of AFB1 are genotoxic) and oncogene activation [34], [35]. We queried the RNA-Seq dataset (containing all possible AFB1-induced gene changes by DESeq) for hub genes which we defined as controlling ≥5 downstream genes that could be directly or indirectly regulated by E2f1 using IPA’s Grow Pathway algorithm (Table S10). We found 223 directly or indirectly affected transcripts (Figure 8) that mapped 198 transcripts that were upregulated and 25 transcripts that were downregulated. The pathway in Figure 8 shows a network of cellular processes potentially influenced by the E2f1 transcription factor, including hub genes for cell cycle control and proliferation (Cdk1, Mdm2, Ect2, Mad2L1, Nuf2, GNAI1), cell death (FAS), cellular damage by electrophiles (Mdm2, Gstp1), growth factors (Fgf1) and tissue remodeling (Mmp2, Ezr, App, Mme). Upregulated genes by DESeq in this integrated pathway of particular interest for hepatocellular proliferation and transformation were follistatin (442-fold), Aldh3a1 (302-fold), Mybl2 (21-fold), Mybl1 (6-fold), and Sox9 (6-fold).

## Discussion

Liver cancer from chemical and viral (e.g. hepatitis C) exposures is still an international problem for which AFB1 is a primary, contributing etiological factor [36], [37], [38]. Dietary models of AFB1 exposure in rodents continue to be useful in studying hepatocellular carcinoma [24]. A major objective of our work was to exploit the wider annotation capabilities of NextGen sequencing for differential gene expression to better understand biological processes leading to AFB1 malignancy. As part of a previous larger study, a microarray analysis had been previously been conducted on the same control and AFB1 liver mRNA samples used to conduct the current RNA-Seq study [25]. We hypothesized that the increased dynamic range and base pair resolution capabilities of RNA-Seq would allow for a more sensitive detection of transcripts, as well as, for the detection of new isoforms and gene products, resulting in an enhanced understanding of the AFB1 liver transcriptome. ‘Within group’ comparisons based on 4 rats per group indicated a high degree of reproducibility among samples. Cufflinks customization was used to assemble transcripts and DESeq and Cuffdiff were used to determine DEGs (≥2X fold; p<0.005) from RNA-Seq data and were compared to similarly filtered microarray DEGs. Using DESeq, a total of 1,026 DEGs were found which were more than 400 transcripts or 63% greater than microarray DEGs. Testing of differential expression by Cuffdiff proved to be more stringent resulting in far fewer DEGs in our study, so we employed a Cufflinks pipeline for reference-based assembly and used the assembled transcript definitions with DESeq for differential expression testing. Although we observed a substantial overlap of DEGs by DESeq and microarray, some DEGs were observed which were unique to either platform. When normalized gene expression of RNA-Seq and microarray data were separated into four quartiles ranked on the basis of average control signal across replicates, the Spearman rank correlation coefficient increased going from lowest to highest expression genes. This indicates that transcripts with low microarray signal or small read counts contribute to the weak correlation between platforms for low expression genes and some of the differences in observed unique DEGs between RNA-Seq and microarray. However, other factors for platform differences are the presence of novel exons and full length transcripts in the rat transcriptome shown by RNA-Seq data which have not yet been annotated by microarray probes. Notably, both platforms easily separated treated animals by PCA and cluster analysis. qPCR validation of eleven selected transcripts covering a wide range of expression showed good agreement in direction and fold-increase for gene expression among expression platforms. Overall, RNA-Seq produced a more comprehensive DEG profile than microarray analysis under the conditions of our study, which was due to measuring reads across the entire transcriptome in agreement with the conclusions of other reports [14], [39], [40].

Representative criteria for DEGs at ≥2-fold differences and p≤0.005 demonstrated areas of overlap and uniqueness between RNA-Seq (DESeq and Cuffdiff) and microarray observed by Venn diagram analysis. Many reasons were found for these differences. For example, the detection of reads from a transcript for which no probe was assigned was a common finding which is understandable considering the more complete mapping provided by RNA-Seq data. RNA-Seq provided data at either the 3′-end or 5′-end of RefSeq or Ensembl transcripts or sometimes in unannotated regions of the transcriptome for which microarray probes did not exist (e.g., Chr8 Cufflinks transcripts in Figure 3D), or had not been assigned any specific annotation (e.g., probes at 3′-Utr of Stxbp5L). Probe placement within specific regions of a transcript also appeared to be a determinant for differential expression since the collective number of RNA-Seq reads per transcript could outweigh a single probe or even multiple probe signals for DEG criteria. For example, 3.7-fold increase in reads across the Srxn1 transcript by DESeq was in contrast to no change in signal by microarray from the probe located within the 3′-UTR. Probe placement and probe annotation as well as the large number of RNA-Seq reads counted across the length of each transcript were factors that greatly influenced the number of DEGs found by RNA-Seq compared to microarray platforms.

Another important aspect of gene expression is the Cufflinks-derived exon expression compared to RefSeq annotations to determine known exons, novel exon boundaries and novel exons. It is important to note that for this analysis we only analyzed those exons that had a significant signal, so that exons with low and possibly irreproducible signal were removed from consideration. Classifications were defined as ‘Exact’ exon matches, ‘Overlapping’ or ‘Within’ matches and ‘Novel’ exons either within (−T) or outside (U-unclassified) RefSeq transcript structure. A majority of the ‘Exact’ matches were found as ‘Common’ to CTRL or AFB1 groups (3650 exons) with many fewer as unique to either treatment. Interestingly, there were many more ‘Overlap’ exons for AFB1 or CTRL groups than were shared (Common). These data likely represent the substantial splicing and differences in exon junctions (accompanying the large concordance of ‘Exact’ exon matches) between treatment groups. For example, nearly 20,000 previously unreported exon junctions were uncovered after annotation-based mapping of mammalian RNA-Seq reads by TopHat [41]. In addition, we also report about 200 shared and unique ‘Novel-T’ exons which were found between exons of known transcripts, such as F11 (Figure 5C), and almost 100 ‘Within’ exons which were also identified. The number of ‘Novel-U’ exons is of particular interest since approximately 1,000 were found for CTRL or AFB1 groups, such as new 5′-exons for ASS1 (Figure 5D), and 179 Novel-U exons which were common to each group.

Ugt1a isoforms represent a specific case posing a challenge for measurement of each isoform using either RNA-Seq or microarray. The UGT1 family consists of thirteen genes that are all five exons in length for which exon 2 to exon 5 are common for all UGT1 mRNAs but the first exon is unique to each gene (UGT1A1 to UGT1A13P) [42]. Since the shared exons comprising each Ugt1a isoform cannot be definitively assigned to any one isoform, exon-specific probes or RNA-Seq signal at each isoform-defining exon would be required for accurate differential expression. Exon-specific reads from RNA-Seq could be measured in our analysis but several Ugt1a isoforms (e.g. Ugt1a7c, Ugt1a5 Ugt1a3 and Ugt1a2) did not have designated microarray probes for measurement. In the present study, we report subchronic AFB1 exposure causes isoform specific upregulation in Ugt1a6 and possibly for Ugt1a7c. Some studies have documented a general increase in Ugt1a expression induced by AFB1 in HepaRG [43] or FLC-4 human hepatocarcinoma cells [44], and in rat liver [45] and more recently, an upregulation of Ugt1a3 mRNA was reported in cultured HepaRG cells after a 48 hr AFB1 treatment [43]. Our results of isoform specific induction from *in vivo* AFB1 exposure could have profound implications for liver function, organ development and toxicity since a dramatic ontogenic isoform switching has been reported in UGT1 isoforms in rats during gestation, infancy, early childhood at days 14–28 and young adulthood at day 56 [46]. It is also worth noting that specialized methods such as CAGE (cap analysis of gene expression) can complement RNA-Seq data in studying genes with multiple isoforms since CAGE selects for 5′-mRNA ends to find exact locations of TSSs (transcription start sites) [47]. Interestingly, after generating human and mouse CAGE libraries it was found that the Ugt1a gene family has seven promoters that are preferentially used by different tissues as well as six alternative ATGs to accommodate individual tissue needs [48].

RNA-Seq also holds intriguing possibilities for novel transcript discovery. Several hundred (observed in two or more animals) to thousands (observed in at least one animal of each group of four animals) of putative, novel transcripts were found in control or AFB1 treated animals depending on the level of replication stringency (Figure 4). The number of novel shared transcripts as ‘AFB1+ Control’ remained higher (927, 479, and 234 novel transcripts, at two, three, and four of four animals, respectively) than transcripts unique to either Control or AFB1 alone at similar levels of replication. These data suggest a limited number of treatment-specific, novel transcripts for follow-up study. We validated two such novel AFB1-responsive transcripts identifying them as HAfT1 and HAfT2 on Chr1.q55 and Chr15.q11, respectively. Four exons comprising an 809 bp cDNA were confirmed by PCR cloning for HAfT1. There is no known prior annotation or EST corresponding to this transcript suggesting it is a completely novel gene about 150 kD downstream from rat 5′-end of the RefSeq gene Tcf7l2 in antisense orientation. This region of rat Chr1 also bears some homology to a portion the first intron of mouse Tcf7l1. A second novel AFB1-responsive gene homologous to one spliced EST (AA851790) was cloned as a 209 cDNA sequence corresponding to the last exon of this proposed 4 exon transcript, made up of two overlapping Cufflinks transcripts (00021611 and 00022036). Ongoing work will clarify the expression and function of these two novel transcripts regarding their upregulated response to AFB1 exposure.

A further objective of our study was to generate new insights into alterations in gene networks that might lead to the formation of hepatocellular carcinomas. The increased sensitivity, wider dynamic range and base-pair resolution profile possible with RNA-Seq compared to microarray platforms reported by others [49], [50], [51] provided a greater biological depth in unraveling changes to the transcriptome due to AFB1 effects. Analysis of DEGs showed activation of various canonical pathways that were involved in xenobiotic metabolism and detoxification, cell cycle alterations, oxidative stress and malignancy signaling. Since canonical pathways can sometimes be overrepresented by similar gene sets [52], we also interpreted transcript interactions and relationships (Figure 7 and accompanying supplemental tables) in light of a perpetual, low level genotoxicity during AFB1 dietary exposure that engages drug elimination enzymes, redox stress and a nascent cell turnover [19], [23], [24]. We found that the pattern of cell cycle transcript upregulation in DEG datasets using RNA-Seq_DESeq shows similarities to low level genotoxic damage described in some rodent liver cancer models; for example, p53 IHC (immunohistochemistry) negative preneoplastic liver lesions stain positive for Gst-P and Mdm2 with either chronic diethylnitrosamine or AFB1 exposure in rats [53] and are often accompanied by increased in Slc7a11 [54], a cystine-glutamate transporter induced during GSH depletion and redox stress [55]. In RNA-Seq data, Gst-P, Mdm2 and Slc7a11 transcripts were elevated by 73-fold, 2-fold and 51-fold, respectively, by AFB1 and p53 was not significantly altered. Similarly, it is not unusual to observe Cdkn1a induction (3-fold) as a protective response to redox stress [56]. In all DEG datasets, the increases in apoptosis-related genes such as Fas (2- fold) were relatively minor as were changes in DNA damage and repair transcripts. Using only transcripts from the DEGs of RNA-Seq in Figure 7, the highest number of interactions were among Cdkn1a, E2f1, Cdk1 and Mdm2 as well as transcripts whose proteins help create the mitotic spindle (Prc1, Racgap1, Plk1, Mad2L1, Spag5) and proteins associated with the kinetochore (Aurkb, Ndc80, Dsn1, Bub1, Nuf2 and Nsl1), the microstructure on chromatids where spindle fibers attach during mitosis. For example, Aurkb controls microtubule dynamics by phosphorylation of the Ndc80 complex, an essential microtubule-binding component of the kinetochore [52] and by phosphorylation of kinetochore component Dsn1 [54]. Furthermore, others have reported that the normal phosphorylation of Aurkb by the mitotic kinase, Bub1, is dramatically increased in Bub1 transgenic mice, causing hyperphosphorylation and increased Aurkb activity [57]. These events cause chromosomal missegregation and aneuploidization leading to formation of multiple spontaneous tumors in Bub1 transgenic mice [57]. Dysregulation of such nuclear structures and associated kinases indicated by RNA-Seq data are consistent with AFB1’s well-known mutagenic and clastogenic [58], [59] properties.

E2f1 is a transcription factor regulating cell-cycle, DNA replication, differentiation, apoptosis and DNA damage [60] and may play a role in liver tumorigenesis [61]. We found E2f1 as a regulatory hub that controls a large number of downstream transcripts (Figure 7) altered by AFB1 exposure which suggested E2f1 could be one of the drivers for cell proliferation and tissue remodeling. We report several E2f1 regulated- and indirectly-regulated transcripts further downstream in Figure 8 which help reinforce AFB1’s effects on mitotic spindle assembly and kinetochore components, implicating Ect2, in addition to those transcripts already identified from Figure 7. Screening microarray data from the NCI-60 cell line panel similarly revealed a common coregulation of E2f1 expression (among other transcription factors) with transcripts involved in either forming kinetochore components and proteins responsible for kinetochore maintenance, including Cenpe, Cenpf and Incenp [56] (each of which were upregulated by AFB1, see Figure 8).

Two DEGs found by RNASeq in E2F1-mediated pathways that were highly upregulated by AFB1, were Wfikkn2 (WAP, follistatin/kazal, immunoglobulin, kunitz and netrin domain containing 2) and an aldehyde dehydrogenase isoform, Aldh3a1. The activin-follistatin system is comprised of members of the TGF-β family for cell growth and differentiation and is critical for maintaining liver homeostasis and in tissue rebuilding and repair [62]. Imbalanced expression of follistatins and activins in preneoplastic foci and hepatoma cells is well known [63] and some researchers suggest follistatin expression is required for proliferation and colony expansion of progenitor populations of hepatocytes [64]. In cultured MEF cells from Mmp2^−\−^ mice, transfected human MMP2 (matrix metalloprotease-2) protein was found to increase the processing of Wiffk2 [65] and researchers have suggested that binding of WFIKKN proteins with these growth factors may localize their action and thus help to establish growth factor gradients in the extracellular space [66]. We identified Mmp2 as a key protein upregulated by AFB1 and its activation is inferred by increased expression of Ddr1 [67]. Upregulated Mmp2 and Ddr1 are events consistent with their respective roles in tissue remodeling in preneoplasia [68], [69]. Additionally, the 2 to 3-fold increase in the gelatinase Mmp2 [70] and the metalloelastase Mmp12 have been linked to invasiveness in rat liver tumor models [71]. Of related interest is that elevations in aldehyde dehydrogenases, in particular Aldh3a1, may play a role in self-protection and expansion of stem cell populations [72]. Upregulation of liver cancer stem markers, Sox9 [73], Epcam [74] and Dmbt1 [75] were observed in this study. It is of interest that Sox9 controls Dmbt1 (deleted in malignant brain tumor 1) expression which is highly amplified during the emergence of ductal (oval) stem cell populations in injured liver [75].

### Conclusions

In conclusion, RNA-Seq analysis demonstrates hundreds of new transcripts and isoforms that includes gene expression changes at a greater dynamic range than microarray and provides deeper insight into pathways and molecular events at an AFB1 exposure level and time prior to the formation of liver tumors. The base pair resolution of RNA-Seq provides an expanded depth of gene expression changes and therefore increased annotation of the rat liver transcriptome. We report novel genes, HAfT1 and HAfT2, and potentially other transcripts that may prove important to chemical response and liver biology. Pathway analysis findings were interpreted as a reflection of a slow hepatocellular turnover supported by an active stem cell population in response to continual, low level, genotoxic injury by AFB1 in the presence of barely perceptible apoptosis. Expression of various ECM proteases suggests a pattern of tissue remodeling and activation of surface receptors that favor tumor development and progression [76]. Differential expression of numerous components and associated kinases of the mitotic spindle assembly and kinetochore structure that support cell division are visible in this type of AFB1 feed exposure model which might otherwise be masked by other high dose, injurious models of chemical carcinogenesis. While the structure of the pathways reported here is contextualized to AFB1 exposure, it could form the basis for further hypothesis testing of other carcinogenic agents for similar transcriptional drivers, signaling pathways, activation of kinases, affected subcellular structures and transcriptional changes within specific areas of the liver architecture. Collectively, these findings highlight the potential contribution of toxicant-mediated perturbations of the transcriptome as a special tool for novel gene discovery using high resolution, NextGen sequencing technologies.

## Materials and Methods

### RNA Extraction, Transcript Profiling and Illumina Sequencing

All experiments were performed in accordance with the Animal Welfare Act and the U.S. Public Health Service Policy on Humane Care and Use of Laboratory Animals after review and approval by the Institutional Animal Care and Use Committee (IACUC) of Battelle Laboratories, Columbus, OH. Male F344/N rats were exposed to 1 ppm AFB1 in feed for 90 days and RNA was obtained from fresh frozen liver as previously described [25]. At necropsy rats were anesthetized with isofluorene, the left and median lobes of the liver were removed and animals were euthanized by exsanguination. A cross-section of each lobe was obtained for histopathology. The remainder of the left and median lobes of the liver were minced quickly into very small pieces and frozen in liquid nitrogen within 4 min of euthanasia and stored at −80°C.

Briefly, RNA was extracted from 130–150 mg of liver tissue with Qiagen RNeasy Midi kits (Valencia, CA, USA). A 500 ng amount of total RNA was converted into labeled cRNA with nucleotides coupled to fluorescent dye Cy3 using the Low RNA Input Linear Amplification Kit (Agilent Technologies, Palo Alto, CA, USA) according to the manufacturer’s protocol. Cy3-labeled cRNA from each sample was hybridized to Agilent Rat Whole Genome Oligonucleotide microarrays in a 4×44 K format. Microarray data are available through the NTP Chemical Effects in Biological Systems (CEBS) database with the accession number, 002-00100-0003-000-6, at the URL site, http://www.niehs.nih.gov/research/resources/databases/cebs/index.cfm and direct access to raw data files are at ftp://157.98.192.110/ntp-cebs/individualstudy/002-00100-0001-000-4/NTP009-Hepatocellular_CarcNon-CarcTox/RawFiles/. (Further details on microarray file access in the CEBS database are in Figure S10.).

For RNA-Seq, RNA libraries were created from each of four controls and four AFB1 treated male F344/N rats. RNA samples were the same as those used for the microarray analysis studies. Starting with 5 µg total RNA, polyA-tailed mRNA was isolated by oligo(dT) and fragmented by adaptive focused acoustic energy (Covaris Inc., MA, USA). A random hexamer primed, cDNA library of nucleotide sequences (400 bp median fragment size) was created from which millions of short DNA reads were generated in a paired-end orientation. Sequencing was performed on eight RNA samples in individual lanes of an Illumina GXIIx instrument (Illumina, San Diego, CA, USA) by the NIH Intramural Sequencing Center (NISC). Each lane produced 29–37 million raw paired reads. Data output in fastq file format contained information about sequences and quality (Phred quality score). Average Phred scores of ≥20 per position were used for alignment.

### Bioinformatic Analysis: Alignment of Paired End Reads

RNA samples were sequenced by the standard Illumina protocol to create raw sequence files (.fastq files) which underwent quality control analysis using FastQC (http://www.bioinformatics.babraham.ac.uk/projects/fastqc/). Two of the eight total samples (one each from CTRL and AFB1 treated samples) were initially sequenced as a pilot experiment at paired end read length of 100 bp each; however they showed declining average base call quality beyond the 75th nucleotide of the 100 bp read. To avoid low quality data negatively influencing downstream analysis, we trimmed the reads on the 3′-end and only used the first 75 bp from the 5′-end of each read for further analysis. The remaining six samples were sequenced at 75 bp paired end read length and did not show any quality issues and hence they were retained at 75 bp length without any trimming. Quality Control (QC) plots are provided in the supplementary information (Figure S1). We aligned the quality checked reads to the Rn4 build of the Rat genome (http://hgdownload.soe.ucsc.edu/goldenPath/rn4/chromosomes/) using TopHat version 1.3.2 (parameters: -g 1 -r 200–best –strata) allowing for unique non-gapped alignments to the genome [27]. We modified the TopHat source code to add the parameters “–best –strata” to the standard set of parameters used.

Resulting alignments were summarized to the evaluate number of uniquely aligned reads per sample along with information such as singleton vs. both-ends mapped, and number of spliced alignments per sample (Table 1). Aligned reads were converted to UCSC genome browser tracks and uploaded to the browser to allow for visual inspection of normalized signal at any genomic location. The UCSC browser tracks contain RPM (Reads Per Million) normalized read counts. Deep sequencing data files are stored in the Sequence Read Archive (SRA) under Study Accession No. SRP017598 that contains sample accession numbers to fastq data files.

### Analysis of Differential Gene Expression

DEG’s were identified using DESeq version 1.8.2 [77] and Cufflinks version 1.3.0 [78], [79]. These methods represent two widely accepted and complementary analysis approaches of RNA-Seq data. For DESeq analysis, we first obtained RefSeq gene annotation for all known genes in the rat genome, as provided by the UCSC genome browser as of Oct 2011. RefSeqGene is a subset of NCBI’s Reference Sequence (RefSeq) project and defines genomic sequences that have sufficient literature support as reference standards for well-characterized genes that generally represent a prevalent ‘standard’ allele. This annotation included 17,194 unique transcript entries with genomic coordinates. Using the reads mapped to the genome, we calculated the number of reads mapped to each transcript in the above RefSeq annotation table. These raw read counts were used as input to DESeq for calculation of normalized signal for each transcript in the CTRL and AFB1 samples, and differential expression was reported as Fold Change along with associated p-values. DESeq calculates p-values using a negative binomial distribution which accounts for technical as well as biological variability. The DESeq approach is well suited for count data (read counts) as is the case for RNA-Seq experiments, and the method estimates variance in a local fashion for varying signal strength [79].

Cufflinks is a complementary method that assembles transcripts and estimates its abundance using read data. Transcript assembly allows for identification of splice variants, new exon boundaries, novel exons, or novel full length transcripts. The differential expression is calculated by Cuffdiff based on transcript abundances [79]. We used Cufflinks v1.3.0 with parameters set at: “–GTF-guide refseq.GTF –frag-bias-correct –multi-read-correct”. The resulting Cufflinks assemblies of all eight samples were combined together using Cuffcompare v1.3.0 with parameters “-r -M –N –s”. Cuffdiff v1.3.0 was then employed on the combined transcripts to identify differentially expressed genes/transcripts with parameters set at “-r –frag-bias-correct –multi-read-correct”.

### Identification of Novel Transcripts

To identify novel transcripts assembled either in the CTRL or AFB1 treated samples, we compared the genomic coordinates of Cufflinks assembled transcripts to known genes annotated in the RefSeq (17,194 unique transcripts) table. If a Cufflinks transcript does not overlap with any known RefSeq transcript (i.e. they occur in intergenic regions without any RefSeq annotation) it was considered to be putatively ‘novel’.

Among those that were considered novel, we further narrowed the list by requiring the putative novel transcript to be present in more than one of the biological replicates. Since the Cufflinks analysis assembled these transcripts individually for each replicate, it is likely that the identical transcript may not be identified in each sample. However by requiring the transcript to be present in at least two of the four biological replicates, we could focus upon reproducibly detected transcripts. Among those that were found in more than one replicate, we further narrowed our list by requiring the novel transcript display statistically significant differential expression (absolute Fold Change >2, p-value<0.005 as calculated using the DESeq method). A table describing differentially expressed Cufflinks transcripts, their normalized signal and fold change values, including those potentially novel ones is provided in the supplemental table information.

### Exon Classification and Annotation

We compared the list of exons obtained from Cufflinks assembled transcripts to all exons present in the RefSeq annotation table. The goal of this comparison was twofold; first, we wanted to identify completely novel exons that were part of previously annotated genes and secondly, we wanted to identify exons where our data indicates partial disagreement with available RefSeq annotation, thus indicating potentially novel exon boundaries for the given exon [27].

Prior to classifying exons, we wanted to ensure to analyze only exons that had a statistically significant signal. To accomplish this, we first calculated the read count per Cufflinks assembled exon, and computed a signal threshold as described below. The threshold to detect significant novel Cufflink exons from each sample was computed as:
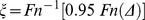



In this equation, *F_n_* denotes the empirical distribution function of read counts from all RefSeq exons where:
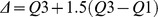



The preceding expression denotes the outlier limit in which *Q_1_* and *Q_3_* represent the first and third quartiles of all unique read counts from all RefSeq exons, respectively.

Following this analysis, we removed exons that were detected as outliers based on their read counts. Using empirical relative frequency distribution of read counts for the remaining exons in each sample, a minimum number of reads were determined at p<0.05 per sample. This criterion was applied to each sample and exons that passed the criteria were used for further exon classification analysis. Further details can be found in Trapnell et al [27].

Cufflinks exons were classified by comparing them to all RefSeq exons. If a Cufflinks exon exactly matched a RefSeq exon (i.e., exon start and end positions are identical), it was labeled as ‘Exact’. If a Cufflinks exon did not overlap a known RefSeq exon by one or more base pairs, and the exon was located in between the transcription start and end site of a gene, we labeled it at as a ‘Novel-T’ exon (T for within a Transcript); otherwise it was labeled as ‘Novel-U’ (U for Unknown). If a Cufflinks exon overlapped with a RefSeq exon such that it was completely contained within the RefSeq exon, it was labeled as ‘Within’. If the Cufflinks exon had partial overlap with the RefSeq exon it was labeled as ‘Overlap’ (for more details refer to supplemental information, Figure S8).

### Mapping of Microarray probes to Cufflinks Transcripts

Comparisons between gene expression values measured by microarray vs. RNA-Seq were performed by first mapping all available microarray probes to their corresponding rat transcripts. The available microarray data was generated in a previous study using Agilent Rat Whole Genome Oligonucleotide microarrays in a 4×44 format, using the identical rat liver RNA from these same samples [25] that were used for RNA-Seq analysis in the current study. To compare signal/fold change obtained from Microarray and RNA-Seq platforms for all possible transcripts from the rat transcriptome, we first created mapping between all Agilent probes to the rat genome (Rn4) using bowtie2 local alignment (parameters –local -M 10 -D 30 -R 10 -N 1) [80]. We used the default mode of bowtie2 where it searches for multiple alignments to the genome and then reports the best scoring local alignment. Using these alignment results, we found 26,310 microarray probes that overlapped exons of Cufflinks transcripts. We used this set of 26,310 probes for further analysis since they directly corresponded to the set of cufflinks transcripts for which fold change/p-values were calculated from RNA-Seq data.

### Microarray Data Normalization

Agilent whole genome microarray data (feature extraction software produced raw microarray signal) for the eight samples were log2-normalized and summarized for each probe using a median polish algorithm. The signal from multiple probes overlapping the same Cufflinks transcript was summarized using a median polish algorithm. Thus, for each Cufflinks transcript that overlapped one or more microarray probes, we obtained a microarray signal and calculated CTRL vs. AFB1 treated fold changes and corresponding p-values. Here the p-value was computed using empirical distribution of Student’s t-statistic derived from 10000 random sample label permutations [81]. To identify differentially expressed genes identified by the microarray study, we employed an absolute Fold Change>2 and p-value <0.005 cutoff uncorrected for multiple testing.

### RNA-Seq Data Normalization

The raw RNA-Seq read counts for Cufflinks transcripts were first log2 transformed at RPM = reads per million and then quantile normalized.

### Combining and Correlating RNA-Seq and Microarray Data

In order to perform correlation analysis on microarray and RNA-Seq data, we used array data from 26,310 microarray probes that map to Cufflinks exons as described above. We used the Cufflinks transcripts that have overlap with one or more microarray probes. The dataset was divided into 4 quartiles ranked on the basis of the average control signal (across replicates) in the microarray platform. Using this microarray and Cufflinks transcript data, we calculated Spearman (Rank) correlation statistics and corresponding asymptotic p-values based on a t-approximation for data points in each quartile. For this particular analysis, we did not correct for transcript length assembled by Cufflinks since our objective was to compute fold differences for each corresponding transcript in Control vs. Treated groups for comparison of transcript expression. This approach allowed us to correlate microarray signal to RNA-Seq signal and obtain a p-value for the correlation.

### Principal Component Analysis (PCA)

We calculated the normalized expression level for all genes in the RefSeq table as implemented in DESeq. We performed PCA using RefSeq gene level signal data for RNASeq_DESeq and microarray platforms. Samples were plotted in three dimensional plots across the first three principal components.

### Pathway Analysis

A list of differentially expressed genes from either the microarray or RNA-Seq platforms was generated using a Fold Change>2, p-value <0.05 criteria and used as an input for Ingenuity Pathway Analysis (IPA) software (licensed use of Ingenuity®Systems, www.ingenuity.com). Canonical pathways that were found to be enriched in the DEG datasets were determined. The significance value associated with overrepresented pathways measures the likelihood that association between an experimental gene set and molecules in Reference gene sets for a specific process or pathway is due to random chance. In general, p-values less than 0.05 indicate a statistically significant, non-random association. The p-value is calculated with the right-tailed Fisher's Exact Test. Ingenuity uses public databases (e.g. HumanCyc) and performs in-house curation to formulate and update signaling pathways. In our study, pathways were constructed with the Build pathways function using either the ‘Connect’ feature for RefSeq annotated DEGs of our microarray, DESeq and Cuffdiff datasets or the ‘Grow’ feature. The Connect feature displayed annotated relationships among DEGs within each dataset, while the Grow feature showed upstream and downstream relationships among DEGs in the DESeq dataset. The ‘Connect’ feature shows relationships among transcripts that have been annotated by Ingenuity®Systems according to the following categories: direct and indirect Interactions, expert and third party Data Sources, various Species, Tissues and Cell lines, Diseases, Molecules (e.g. chemicals and pharmaceuticals) and Biofluids. A pathway of ‘Connect’ relationships was constructed by applying these annotated categories for each DEG dataset from DESeq, microarray and CuffDiff. Hub genes (bolded, enlarged gene symbols in pathway) were defined as those transcripts showing regulation or interaction with equal to or more than five other transcripts. The ‘Grow’ feature uses a specified transcript of interest as a starting point to find relationships (upstream, downstream, direct and indirect relationships) between that transcript and other molecules of interest. The E2f1 pathway was constructed by the ‘Grow’ feature using only the DEG dataset from DESeq analysis, filtered by direct and indirect Interactions from all molecules that are upstream or downstream on specified transcripts. ‘Grow’ started on the E2f1 transcript and we then tested subsequent downstream DEGs in which we defined regulatory transcripts (bolded, enlarged gene symbols in pathway) as those with equal to or greater than five connections (interactions or relationships). Objects representing upregulated genes (e.g. Figures 7, 8) are colored red and downregulated genes are colored green for which increasing color intensity is associated with increasing fold change.

### qPCR Validation of Gene Expression

We have previously validated differential transcript expression of Adam8, Ddit4l Cdh13 Abcb1b Grin2c, Mybl2, Abcc3, Akr7a3, Akr7a2, Cxcl1 and Wwox in the liver of AFB1-treated male rats [26]. qPCR analysis was performed on an ABI Model 7500 Real-Time instrument (Applied Biosystems, Foster City, CA, USA). SuperScript II First Strand cDNA system (Invitrogen, Carlsbad, CA, USA) was mixed with RNA from fresh frozen samples for reverse transcription using random hexamers. Gene changes were determined using the 2^−ΔΔCt^ method by normalizing to β-actin expression which did not vary significantly with AFB1 treatment. Primers and further details are provided in prior work [26].

### PCR Amplification, Cloning and Sequencing

Two potentially novel genes were investigated on chromosome 1.q55 and 15.q11. Based upon RNA-Seq data, primers (Figure S7) were designed for PCR amplification of products from liver cDNA created from Oligo(dT)-primed reverse transcription reactions of liver RNA isolated from AFB1 treated rats. PCR products were gel-purified, cloned and Sanger sequenced. Briefly, amplicons were gel purified in 2% agarose, cut out, melted and purified on silica gel spin columns (Qiagen, Valencia, CA, USA) and TOPO TA cloned into chemically-competent *Escherichia coli* (TopTen cells, Invitrogen) according to the manufacturer’s protocol. Transformed cells were selected for positive clones on 50 µg/mL Kanamycin LB agar dishes and screened for inserts by agarose gel electrophoresis prior to Sanger sequencing of plasmids using forward and reverse M13 sequencing primers (Forward: GTAAAACGACGGCCAG; Reverse: CAGGAAACAGCTATGAC). At least 4 sequences were obtained for each amplicon from two different animals.

## Supporting Information

Figure S1 **Quality assessment of sequencing paired-end RNA-Seq reads from rat RNA.**
(DOCX)

Figure S2 **Nucleotide composition of sequencing paired-end RNA-Seq reads from rat RNA.**
(DOCX)

Figure S3 **Pearson correlation coefficient (r^2^) of within group sample comparisons by Cufflinks transcript level from RNA-Seq analysis.**
(DOCX)

Figure S4 **Pearson correlation coefficient (r^2^) of within group sample comparisons by RMA normalized probe set intensities from microarray analysis.**
(DOCX)

Figure S5 **Comparison of RNA-Seq and microarray data by Spearman correlation coefficient (r_s_) of each sample within control animals.**
(DOCX)

Figure S6 **Comparison of RNA-Seq and Microarray data by Spearman correlation coefficient (r_s_) of each sample within Aflatoxin B1 treated animals.**
(DOCX)

Figure S7 **Novel Transcripts HAfT1 and HAfT2.**
(DOCX)

Figure S8 **Types annotated and unannotated exons assembled by Cufflinks in reference to a model gene.**
(DOCX)

Figure S9 **Examples of Eight Novel Exons Found in Known RefSeq Genes.**
(DOCX)

Figure S10 **Microarray data file access in the CEBS database.**
(DOCX)

Table S1 **DEGs found by DESeq, Microarray, Cuffdiff analysis.**
(XLSX)

Table S2 **Top 30 overexpressed transcripts by DESeq, Microarray, Cuffdiff analysis.**
(XLSX)

Table S3 **FPKM Normalization.**
(XLSX)

Table S4 **Genomic location of 49 novel AFB1 DEGs.**
(XLSX)

Table S5 **Genomic location of novel exons.**
(XLSX)

Table S6 **Common canonical pathways of DESeq, Microarray, Cuffdiff analysis.**
(XLSX)

Table S7 **DESeq connectivity gene pathway.**
(XLSX)

Table S8 **Microarray connectivity pathway.**
(XLSX)

Table S9 **Cuffdiff connectivity pathway.**
(XLSX)

Table S10 **E2f1 connectivity pathway.**
(XLSX)
