# Supplementary material for: RNA-Seq Profiling Reveals Novel Hepatic Gene Expression Pattern in Aflatoxin B1 Treated Rats
Source: PLoS One. 2013 Apr 22;8(4):e61768. doi: 10.1371/journal.pone.0061768 (PMC3632591; doi:10.1371/journal.pone.0061768)
Supplement: Figure S1 — Quality assessment of sequencing paired-end RNA-Seq reads from rat RNA. (DOCX) [file pone.0061768.s001.docx]

**Figure S-1.** Quality assessment of sequencing paired-end RNA-Seq reads from rat RNA.


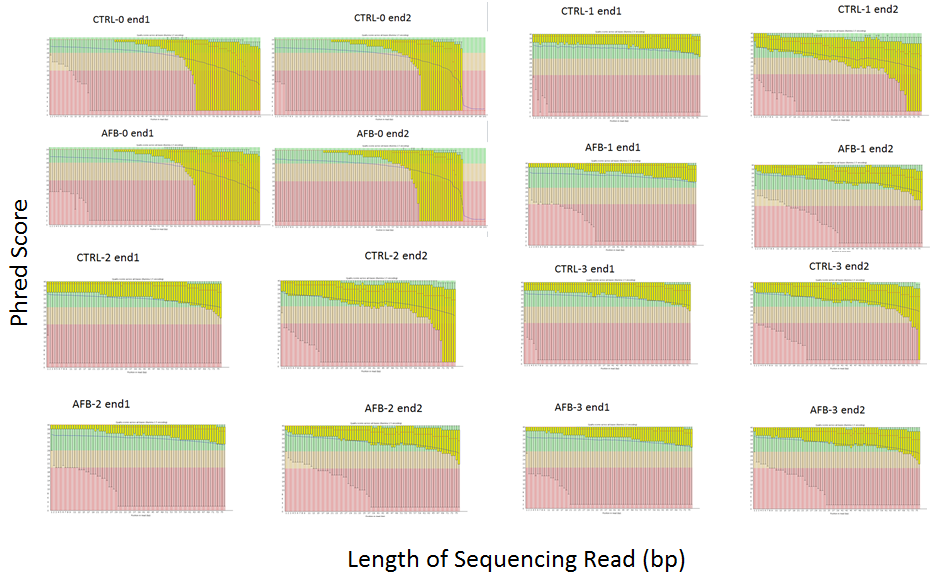


Phred quality scores of RNA-Seq reads were plotted (yellow bars) in each panel for each end of control (CTRL) or aflatoxin (AFB1) treated rats. The range of Phred scores on the y-axis are grouped by color; pink is 0-20, tan is 20-28 and green is 28-36 and the actual scores for each sample are in yellow. Read length is shown on the x-axis (0 to 100, left to right). For the samples, CTRL-0 and AFB-0, 100bp paired-end reads showed that Phred scores dropped below 20 at read lengths greater than 75bp. When the remaining six samples, CTRL-1,2,3 and AFB-1,2,3 were sequenced, all base positions were of good quality (Phred score >20).
