# Supplementary material for: RNA-Seq Profiling Reveals Novel Hepatic Gene Expression Pattern in Aflatoxin B1 Treated Rats
Source: PLoS One. 2013 Apr 22;8(4):e61768. doi: 10.1371/journal.pone.0061768 (PMC3632591; doi:10.1371/journal.pone.0061768)
Supplement: Figure S2 — Nucleotide composition of sequencing paired-end RNA-Seq reads from rat RNA. (DOCX) [file pone.0061768.s002.docx]

**Figure S-2.** Nucleotide composition of sequencing paired-end RNA-Seq reads from rat RNA.


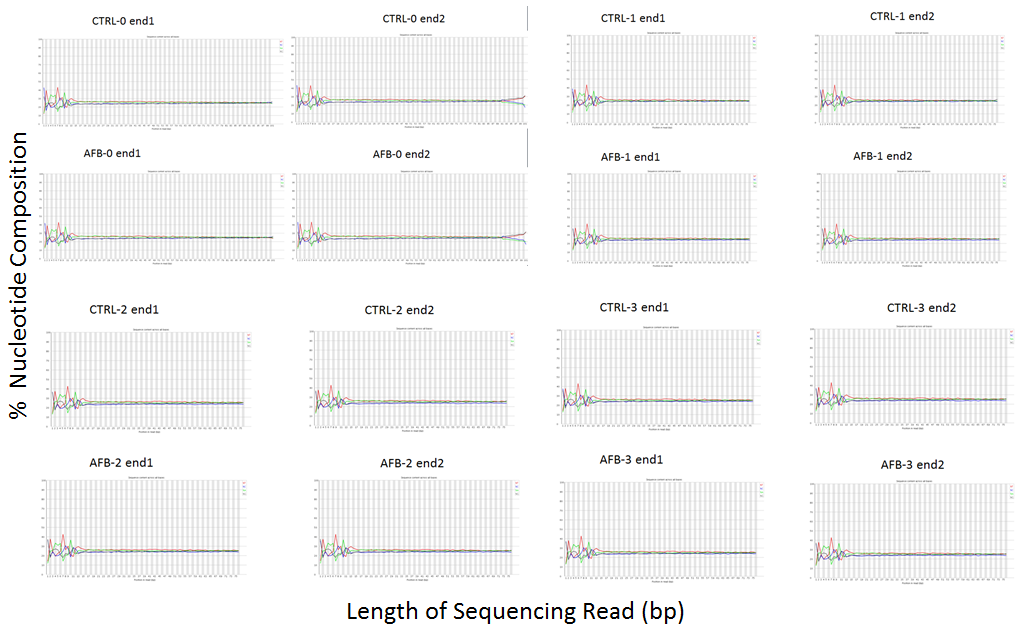


The nucleotide compositions of RNA-Seq reads were plotted (yellow bars) in each panel for each end of control (CTRL) or aflatoxin (AFB1) treated rats. The percentage of each nucleotide (y-axis) is indicated by color (T= red; C = blue; C= green; G = black) and is plotted against read length (x-axis). For the samples, CTRL-0 and AFB-0, the read length extends to 100bp while the remaining six samples, CTRL-1,2,3 and AFB-1,2,3, were at 75bp length. Note the changing composition in beginning reads (1-12) becomes evenly distributed in longer read lengths which is common in RNA-Seq data.
