# Supplementary material for: RNA-Seq Profiling Reveals Novel Hepatic Gene Expression Pattern in Aflatoxin B1 Treated Rats
Source: PLoS One. 2013 Apr 22;8(4):e61768. doi: 10.1371/journal.pone.0061768 (PMC3632591; doi:10.1371/journal.pone.0061768)
Supplement: Figure S3 — Pearson correlation coefficient (r2) of within group sample comparisons by Cufflinks transcript level from RNA-Seq analysis. (DOCX) [file pone.0061768.s003.docx]

**Figure S-3.** Pearson correlation coefficient (r^2^) of within group sample comparisons by Cufflinks transcript level from RNA-Seq analysis.


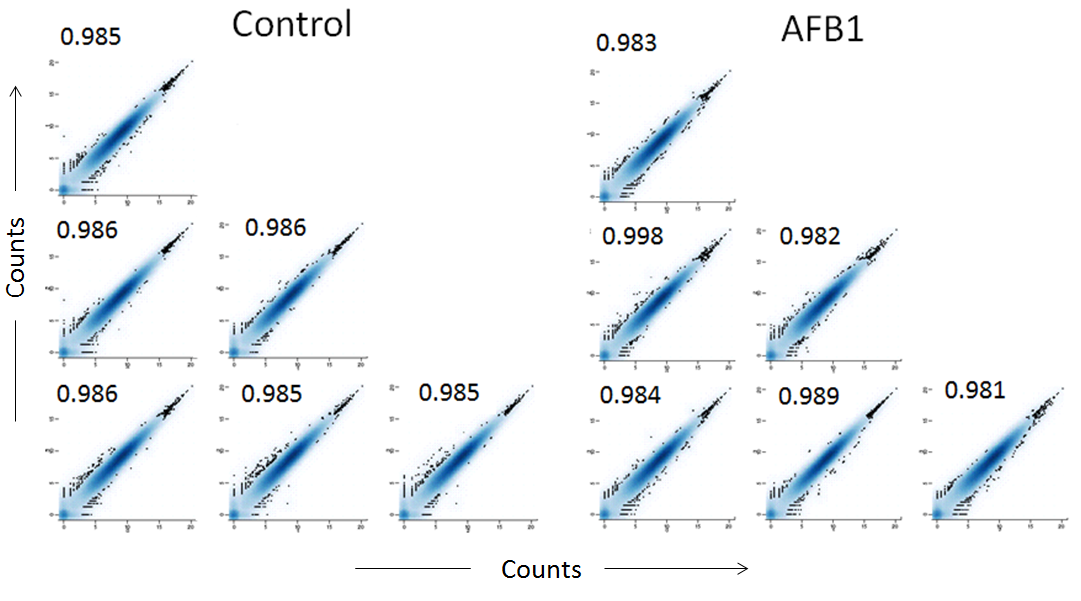


Comparison of gene expression between replicate liver samples of control and Aflatoxin B1 (AFB1) treated rats (n=4/group) was performed after RNA-Seq analysis. Normalized transcript levels were log2 transformed and plotted against transcript levels of other animals within the group. The large correlation coefficients show high degree of reproducibility of data when individual animals were compared within each group.
