# Supplementary material for: RNA-Seq Profiling Reveals Novel Hepatic Gene Expression Pattern in Aflatoxin B1 Treated Rats
Source: PLoS One. 2013 Apr 22;8(4):e61768. doi: 10.1371/journal.pone.0061768 (PMC3632591; doi:10.1371/journal.pone.0061768)
Supplement: Figure S5 — Comparison of RNA-Seq and microarray data by Spearman correlation coefficient (rs) of each sample within control animals. (DOCX) [file pone.0061768.s005.docx]

**Figure S-5.** Comparison of RNA-Seq and microarray data by Spearman correlation coefficient (r_s_) of each sample within control animals.


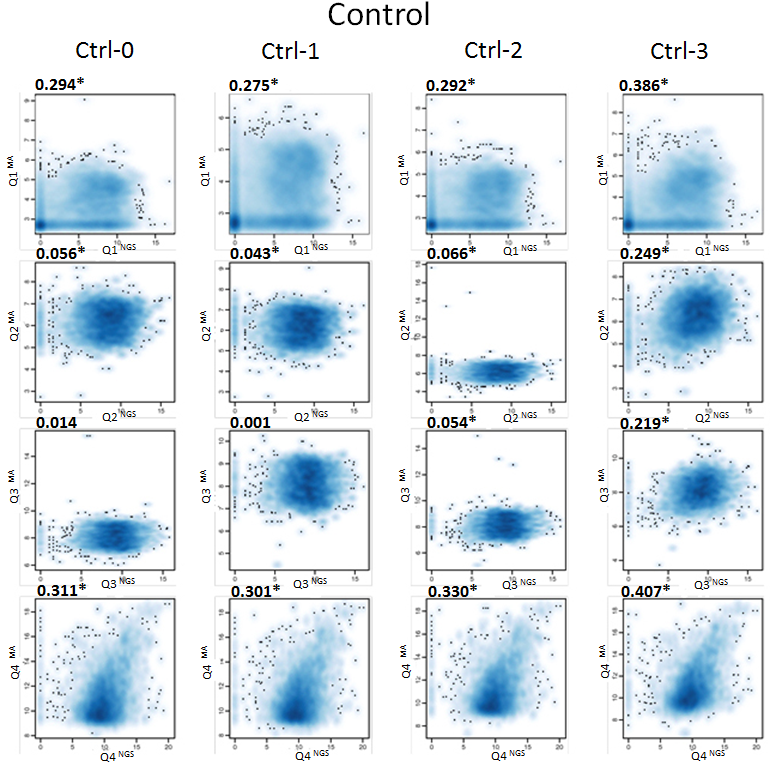


Correlation of transcript expression between RNA-Seq (NextGen Sequencing, NGS; X-axis) and microarray (Microarray, MA; Y-axis) analysis in control (no treatment) animals. Each transcript was plotted as an x,y coordinate with a log2-transformed, quantile normalized RNA-Seq value on the x-axis and a log2 transformed, quantile normalized microarray value of that transcript on the y-axis. Expression of transcripts ranged from lowest in quartile 1 (Q1) to highest expression in quartile 4 (Q4). RNA-Seq data (NGS, Next Generation Sequencing) was first normalized by log2 transformation of raw read counts for Cufflinks assembled transcripts, and then quartile normalized (e.g. X-axis, Q1^NGS^ to Q4^NGS^ for each quartile plot). Note that the x-axis scale is 0 - 15 for Q1, Q2, Q3, and is 0 – 20 for Q4. Microarray data (Microarray, MA) was normalized by feature extraction of the raw microarray signal followed by log2-normalization and summarization for each probe using a median polish algorithm (e.g. Y-axis, Q1^MA^ to Q4^MA^ for each quartile plot). Note that the y-axis scale for log2 values varies slightly for each quartile in order to fit in all data points; approximately 3- 9 for Q1 and Q2; 6-10 or 6-14 for Q3, and 6-14 for Q4. The signal from multiple probes overlapping the Cufflink was summarized using median polish algorithm. Thus, for each Cufflinks transcript that overlapped one or more microarray probes, the microarray signal, AFB1 vs. control fold and p value was obtained. In order to combine RNA-Seq and microarray data, only those Cufflinks transcripts were used that had non-zero RNA-Seq signal and overlapped with 1 or more microarray probes. The data was divided into 4 quartiles and ranked on the basis of the average control signal (across replicates) in the microarray platform. The Spearman (Rank) correlation statistic (bold number at upper left of each quartile comparison), and corresponding asymptotic p-values (* indicates p<0.001) based on t-approximation were computed.
