# Supplementary material for: RNA-Seq Profiling Reveals Novel Hepatic Gene Expression Pattern in Aflatoxin B1 Treated Rats
Source: PLoS One. 2013 Apr 22;8(4):e61768. doi: 10.1371/journal.pone.0061768 (PMC3632591; doi:10.1371/journal.pone.0061768)

**Figure S-7.** Novel Transcripts HAfT1 and HAfT2.

**Panel A.** HAfT1 (Hepatic aflatoxin-responsive transcript 1) on Chr1q55 is from the assembled transcript, Cufflinks_00006229; cDNA sequence from PCR products (primers), predicted amino acid sequence and BLAT alignment of cloned cDNA sequence with the Cufflinks transcript. See text in manuscript for further details.

cDNA 809bp

caGTGTCCTT TCCTGGAACT GCCCCTTGGC ATGAACTCCT GTAGGTGTTC 50

CGTGGGCAGC TGGGTATATA GTTCGGCGGC AGAATGCTTG CCTAGCATGC 100

ACAACGCCTT GAGTTCTGTT CCCAGGGCTG CGTATACCAG GTGTGGTGAT 150

GCACAGCTGT GGCCCCAGCT GGGGAAGCCA AAGAAGAATT TCAAGGTCAT 200

CAATTGCTAC ATCACAAGCT TAACCTGGCT TGATTCATCA ACAAGCCAAT 250

GGCTCATGGA ATGGCGTTGC TGGCCCCTAA GTGATCACAA GACGAGCAGG 300

CTGGAATGTC TAGAAAaAGA GGAAACAGCT GACAGAGTTC CCGCTTTGAC 350

GTCCTGCCCT GATTTTCCTC ACGGATGACT GCGACCTGGA GCTGTGACCT 400

GCGGGGTGCA TGCAGGAGCA GCCGGACTCT CTTCCTCAGT GTGGTACCTT 450

ATCCTCCAGT AAACACTTCC TCTCTAGAAT TAGTGAGTGA GGCCAGCAAG 500

AGAAAGACAT CTTTGGACAA GCGATTTTCA ATTTTTCCCC TCTGCTTCAC 550

ATTCACCAAT GCCCAAGGCA CTAATTCAAG AGCAGGAGGA AGGTTTCGTC 600

TTGGTGAAGT ATCTGCCTCA CAGGAGTAGG GAAGGAAGGA AGGAAGGACA 650

CTTGCATGTC TCTGGGGTGT ATCACAATGT CTCCTCCACT CAGCTGACCT 700

GGCGTTCACG ACTGCCCGAT GATGGGCCCT CGTTCCCACT CTGTGGATGG 750

AGACgGGAAT AGGGAGAAGA CAcCAGAAGC CCCATTGTTT TAGCAGATGA 800

CAAGGGCAG

Primer Set#1 Forward: 5’-GTG TCC TTT CCT GGA ACT GC-3’

Reverse: 5’-CTT TCT CTT GCT GGC CTC AC-3’

*503 bp product*

Primer Set#2 Forward: 5’-GTG TCC TTT CCT GGA ACT GC-3’

Reverse: 5’-ATC ACT TAG GGG CCA GCA AC-3’

*282 bp product*

Primer Set#3 Forward: 5’-TAT ACC AGG TGT GGT GAT GC-3’

Reverse: 5’-GCC CTT GTC TTC TGC TAA AA-3’

*674 bp product*

115 AA predicted translation from predicted ATG start site.

M N S C R C S V G S W V Y S S A A E C L P S M H N A L S S V P R A A Y T R C G D A Q L W P Q L G K P K K N F K V I N C Y I T S L T W L D S S T S Q W L M E W R C W P L S D H K T S R L E C L E K E E T A D R V P A L T S C P D F P H G


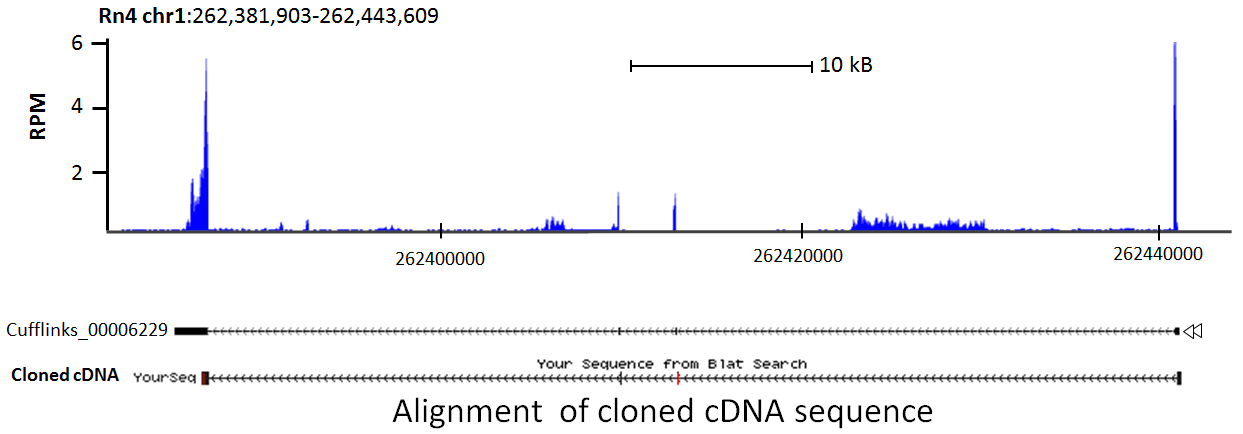


**(Figure S-7, cont’d.)**

**Panel B.** HAfT2 (Hepatic aflatoxin-responsive transcript 1) on Chr1q55 as a composite assembly of transcripts, Cufflinks_0021611 and Cufflinks 22036**;** cDNA sequence from PCR product (primers), and BLAT alignment of cloned cDNA sequence with ESTs and Cufflinks transcripts. See text in manuscript for further details.

cDNA 209bp

TCCCATTACAGATGGTTGTGAGTCGACATGTGGTTGCTGAGAATTGAACTCAGGACGTCTGGAAGAGCAGTCAGTGCTCTTAGCTGCTGAGTCATCTCTCCAGCCCTCATTTATTCTTTTATTCTTTGAGTGTGCACATGGTGTGGCTTCTCCGTGAAGGTCAGAGGCCAGCTTGCGGGCGGGTGGTGACTCTCTCCTTCAGACGCATA

Primer Set#1 Forward: 5’-TCC CAT TAC AGA TGG TTG TG-3’

Reverse: 5’-GAA TAA ATG AGG GCT GGA GA-3’

*115 bp product*

Primer Set#2 Forward: 5’-ACT CAG GAC GTC TGG AAG AG-3’

Reverse: 5’-ATG CGT CTG AAG GAG AGA GT-3’

*180 bp product*


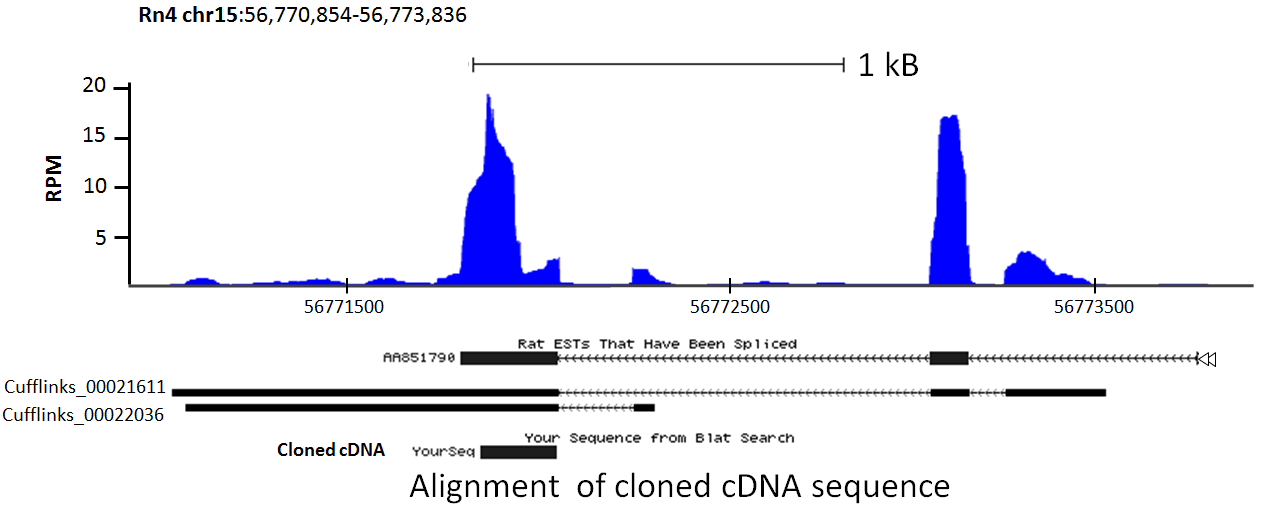

Supplement: Figure S7 — Novel Transcripts HAfT1 and HAfT2. (DOCX) [file pone.0061768.s007.docx]
