# Supplementary material for: RNA-Seq Profiling Reveals Novel Hepatic Gene Expression Pattern in Aflatoxin B1 Treated Rats
Source: PLoS One. 2013 Apr 22;8(4):e61768. doi: 10.1371/journal.pone.0061768 (PMC3632591; doi:10.1371/journal.pone.0061768)

**Figure S-8.** Types annotated and unannotated exons assembled by Cufflinks in reference to a model gene.


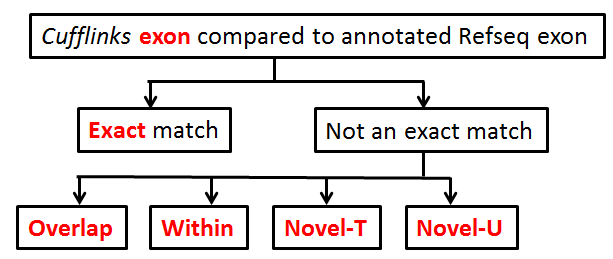


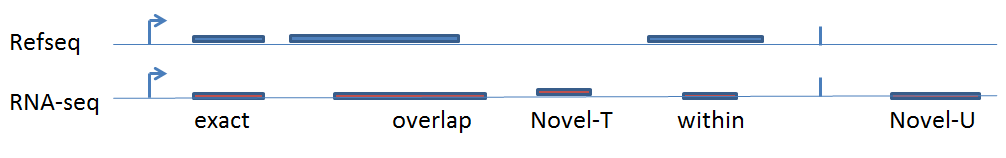

Supplement: Figure S8 — Types annotated and unannotated exons assembled by Cufflinks in reference to a model gene. (DOCX) [file pone.0061768.s008.docx]
