# Supplementary material for: RNA-Seq Profiling Reveals Novel Hepatic Gene Expression Pattern in Aflatoxin B1 Treated Rats
Source: PLoS One. 2013 Apr 22;8(4):e61768. doi: 10.1371/journal.pone.0061768 (PMC3632591; doi:10.1371/journal.pone.0061768)
Supplement: Figure S9 — Examples of Eight Novel Exons Found in Known RefSeq Genes. (DOCX) [file pone.0061768.s009.docx]

**Figure S-9.** Examples of Eight Novel Exons Found in Known RefSeq Genes

The following Figures 9A to 9H show UCSC gene browser examples of Novel exons discovered in known RefSeq genes using the AFB1 RNA-Seq data. These example exons were found to be conserved in known Non-Rat RefSeq or Ensembl genes.

Figure S-9A

Exon not present in Rn4 RefSeq or Ensembl annotation of the gene ADK (adenosine kinase). It is present in known human as well as mouse RefSeq gene annotations.

Figure S-9B

Exon not present in rn4 RefSeq or Ensembl annotation of the gene Asgr1 (asialoglycoprotein receptor 1). It is present in known human as well as mouse RefSeq gene annotations.

Figure S-9C

Exon not present in Rn4 RefSeq or Ensembl annotation of the gene C8g (complement component 8, gamma polypeptide). It is present in known human as well as mouse RefSeq gene annotation.

Figure S-9D

No known RefSeq or Ensembl Rn4 gene annotation have been reported for these exons in this assembled transcript. This is a “novel” transcript assembled by Cufflinks. The novel transcript overlaps known human and mouse RefSeq gene annotations (e.g. Mus homolog for Abca6; ATP-binding cassette, sub-family A (ABC1), member 6)

Figure S-9E

No known RefSeq or Ensembl Rn4 gene annotation have been reported for these exons in this assembled transcript. This is a “novel” transcript assembled by Cufflinks. The novel transcript overlaps known human and mouse RefSeq gene annotations (e.g. mouse homolog for Erbb2IP; erbb2 interacting protein).

Figure S-9F

No known RefSeq or Ensembl Rn4 gene annotation have been reported for these exons in this assembled transcript. This is a “novel” transcript assembled by cufflinks. The novel transcript overlaps known mouse RefSeq gene annotations (e.g. human homolog to OIP5-AS1 human homolog**; OIP5** antisense RNA 1).

Figure S-9G

No known RefSeq or Ensembl Rn4 gene annotation have been reported for these exons in this assembled transcript. This is a “novel” transcript assembled by Cufflinks. The novel transcript overlaps known human and mouse RefSeq gene annotations (e.g. mouse homolog to RPS27L; ribosomal protein S27-like).

Figure S-9H

No known RefSeq or Ensembl Rn4 gene annotation have been reported for these exons in this assembled transcript. This is a “novel” transcript assembled by cufflinks. The novel transcript overlaps known mouse RefSeq gene annotations (e.g. mouse homolog to AW112010; small secreted protein interferon-induced).
