# Supplementary material for: RNA-Seq Profiling Reveals Novel Hepatic Gene Expression Pattern in Aflatoxin B1 Treated Rats
Source: PLoS One. 2013 Apr 22;8(4):e61768. doi: 10.1371/journal.pone.0061768 (PMC3632591; doi:10.1371/journal.pone.0061768)
Supplement: Figure S10 — Microarray data file access in the CEBS database. (DOCX) [file pone.0061768.s010.docx]

**Figure S-10.** Access to microarray files in the CEBS database.

Raw data files can be viewed at the FTP site (below) within the CEBS database by searching the list using the Hyb Name in the table below:

<ftp://157.98.192.110/ntp-cebs/individualstudy/002-00100-0001-000-4/NTP009-Hepatocellular_CarcNon-CarcTox/RawFiles/>

Sample RNASeq Identifier

from Table 1. Treatment Hyb Name

CTRL_0 Control 0ppm 91-day Rat 13 US23502387_251487910428_S01_GE1-v5_91_0806_1_4

CTRL_1 Control 0ppm 91-day Rat 14 US23502387_251487910645_S01_GE1-v5_91_0806_1_1

CTRL_2 Control 0ppm 91-day Rat 15 US23502387_251487910645_S01_GE1-v5_91_0806_1_2

CTRL_3 Control 0ppm 91-day Rat 17 US23502387_251487910645_S01_GE1-v5_91_0806_1_4

AFB1_0 Aflatoxin B1 1ppm 91-day Rat 113 US23502387_251487910646_S01_GE1-v5_91_0806_1_2

AFB1_1 Aflatoxin B1 1ppm 91-day Rat 114 US23502387_251487910491_S01_GE1-v5_91_0806_1_3

AFB1_2 Aflatoxin B1 1ppm 91-day Rat 115 US23502387_251487910646_S01_GE1-v5_91_0806_1_4

AFB1_3 Aflatoxin B1 1ppm 91-day Rat 117 US23502387_251487910492_S01_GE1-v5_91_0806_1_1

Additional sample data can be derived from the instructions below. You will be guided to the FTP Directory [**NTP009-Hepatocellular_CarcNon-CarcTox**](ftp://157.98.192.110/ntp-cebs/individualstudy/002-00100-0001-000-4/NTP009-Hepatocellular_CarcNon-CarcTox/) at the URL site, <ftp://157.98.192.110/ntp-cebs/individualstudy/002-00100-0001-000-4/NTP009-Hepatocellular_CarcNon-CarcTox/> that contains a ‘RawFiles’ folder containing the relevant microarray data files with the Hyb Name in the above Table.

1. Go to the CEBS website at:

<https://www.niehs.nih.gov/research/resources/databases/cebs/index.cfm>

1. Click on Open CEBS


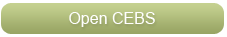


1. Click on CEBS accession number


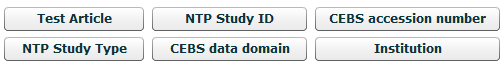


1. Enter the accession number: 002-00100-0003-000-6; and click on Search to find the study folder.


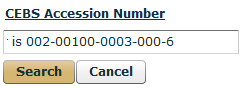


1. A small grey arrow at the left points to the study folder icon. Click on the grey arrow (see below) so it points downward and reveals the desired data folder 002-00100-0003-000-6.


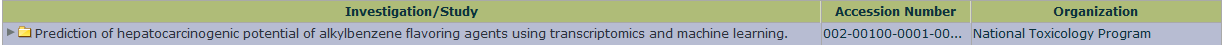


1. Click on the data folder labeled, ‘Toxicogenomic Evaluation of Rat Liver Carcinogens…’


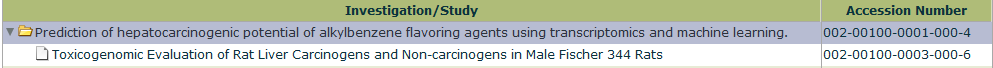


1. Double click on the data fold icon, ‘Toxicogenomic Evaluation of Rat Liver Carcinogens…’ to reveal Characteristics of data folder page below.


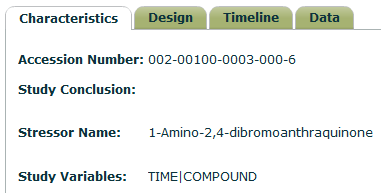


1. Click on the ‘Data’ Tab to reveal Available data page below.


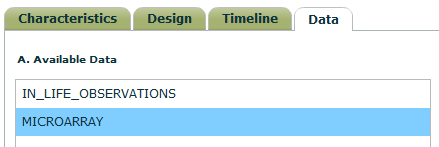


1. Double click on ‘Microarray’ to reveal list of Agilent microarray data files below.


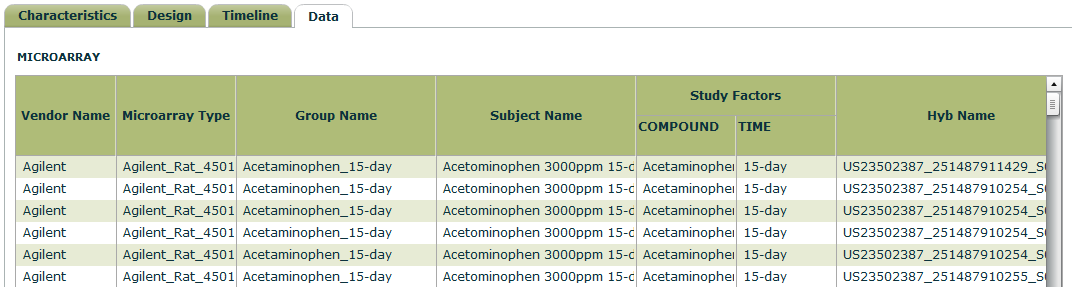


1. Scroll down to the files with the Subject Name and Hyb Name and additional information on each sample.

Sample RNASeq Identifier

from Table 1. Treatment Hyb Name

CTRL_0 Control 0ppm 91-day Rat 13 US23502387_251487910428_S01_GE1-v5_91_0806_1_4

CTRL_1 Control 0ppm 91-day Rat 14 US23502387_251487910645_S01_GE1-v5_91_0806_1_1

CTRL_2 Control 0ppm 91-day Rat 15 US23502387_251487910645_S01_GE1-v5_91_0806_1_2

CTRL_3 Control 0ppm 91-day Rat 17 US23502387_251487910645_S01_GE1-v5_91_0806_1_4

AFB1_0 Aflatoxin B1 1ppm 91-day Rat 113 US23502387_251487910646_S01_GE1-v5_91_0806_1_2

AFB1_1 Aflatoxin B1 1ppm 91-day Rat 114 US23502387_251487910491_S01_GE1-v5_91_0806_1_3

AFB1_2 Aflatoxin B1 1ppm 91-day Rat 115 US23502387_251487910646_S01_GE1-v5_91_0806_1_4

AFB1_3 Aflatoxin B1 1ppm 91-day Rat 117 US23502387_251487910492_S01_GE1-v5_91_0806_1_1
